# Secondary-Sphere Control of Electronic Structure and Ligand Basicity in Mo-Nitrogenase: A QM/MM Analysis Guided by Se K‑Edge HERFD XAS

**DOI:** 10.1021/jacs.6c16459

**Published:** 2026-10-20

**Authors:** Gursahib Singh Sethi, Justin P. Joyce, Christina Roemelt, Kushal Sengupta, Serena DeBeer

**Affiliations:** Department of Inorganic Spectroscopy, 28313 Max Planck Institute for Chemical Energy Conversion, Stiftstr. 34-36, Mülheim an der Ruhr D-45470, Germany

## Abstract

Molybdenum nitrogenase catalyzes biological nitrogen fixation using a complex metallocofactor (FeMoco) containing three μ_2_-“belt”sulfides that exhibit distinct reactivity during catalysis. In particular, protonation at the S2B site imparts lability that facilitates substrate binding and may enable this sulfide to act as a proton donor. Although FeMoco is inactive outside its protein environment, how secondary-sphere residues tune its geometric and electronic structure to enable function remains unclear. We have previously shown that site-selective Se substitution of the μ_2_-sulfides provides a direct probe of the ligand environment using Se K-edge high-energy-resolution fluorescence-detected X-ray absorption spectroscopy (HERFD XAS). Despite the cofactor’s approximate *C*_*3*_ symmetry, Se-substituted sites display pronounced differences in Se HERFD XAS pre-edge intensities. Utilizing QM/MM calculations, we show that hydrogen-bonding (H-bonding) residues polarize the S3A and S5A sulfides, reducing metal–ligand covalency and attenuating pre-edge intensity, whereas hydrophobic residues surrounding S2B localize electron density and enhance its pre-edge intensity. These results demonstrate how hydrophobicity and H-bonding within the secondary coordination sphere modulate FeMoco electronic structure. More broadly, they show how the protein environment tunes ligand basicity to favor selective S2B protonation and provide design principles for controlling enzymatic function.

## Graphical abstract

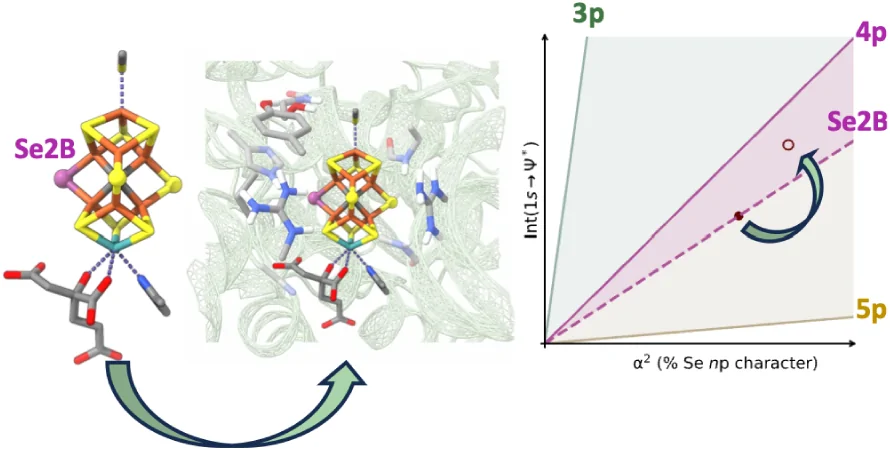

## 1 Introduction

Nitrogenase is the only enzyme known to catalyze the reduction of atmospheric dinitrogen (N_2_) to ammonia (NH_3_) under ambient conditions.^1–4^ This reaction represents one of the most challenging transformations in chemistry due to the extraordinary stability of the N≡N triple bond. Despite decades of investigation, the detailed mechanism of nitrogen reduction by nitrogenase remains incompletely understood. At the center of this catalytic process lies the iron–molybdenum cofactor (FeMoco), a highly complex metal–sulfur cluster where substrate binding and reduction occur. Three naturally occurring nitrogenase isozymes have been identified: the molybdenum-dependent, vanadium-dependent, and iron-only nitrogenases. Each isozyme contains a homologous metal cluster of the general composition [MFe_7_S_8_XC-(*R*)-homocitrate], where the heterometal M can be Mo, V, or Fe and X is a μ_2_-bridging sulfide or a carbonate.^1,2,4^ Among these variants, Mo-nitrogenase is the most efficient at reducing N_2_ to NH_3_, whereas the alternative V- and Fe-only nitrogenases display enhanced activity toward other substrates such as CO and CO_2_.^1–4^ Understanding how the structure of these cofactors governs catalytic reactivity remains a central challenge in bioinorganic chemistry.^1–4^

The FeMoco cluster of Mo-nitrogenase represents one of the most structurally complex metal cofactors found in biology. High-resolution crystallographic and spectroscopic studies have revealed that the cluster consists of two fused heterometallic cubanes bridged by three μ_2_-sulfide ligands and centered around a μ_6_-carbide atom (Figure 1).^5,6^ The cluster is coordinated to the protein through only two amino-acid ligands: the imidazole side chain of His442 (α subunit numbering, *Azotobacter vinelandii* *)*, which binds the Mo center, and the thiolate of Cys275, which coordinates the apical Fe atom (Figure 1, left). These two residues represent the only direct protein linkages to the cofactor, leaving the majority of the cluster embedded within a cavity defined by the surrounding protein matrix. A striking structural feature of FeMoco is the presence of three μ_2_-sulfide ligands that form a belt around the cluster. These belt sulfides, denoted S2B, S3A, and S5A, connect the two cubane subunits. Although these sulfides are chemically similar, crystallographic studies suggest that they play distinct roles during catalysis.^7–10^ In particular, recent studies have proposed that the first protonation event of the Lowe-Thorneley catalytic cycle occurs preferentially at the S2B belt sulfide.^11–13^ This prediction implies that the three belt sulfides are electronically inequivalent despite their similar structural environments. Several factors may contribute to this asymmetry. One important influence arises from the surrounding protein environment. Amino acid residues located near the belt sulfides generate a complex electrostatic landscape that can modulate the electronic structure of the cluster.^14–16^ For example, His195,^17–21^ Phe381, and Val70 ^22,23^ are positioned near the S2B site, whereas the positively charged residues Arg96 ^24^ and Arg359 are located close to the S5A sulfide (Figure 1, right). In contrast, the S3A position is primarily surrounded by backbone amide groups that may form H-bonding interactions with the sulfide. Mutagenesis studies have demonstrated that several of these residues strongly influence catalytic activity, indicating that the secondary coordination sphere plays an important role in controlling FeMoco reactivity.^17–24^ Moreover, the importance of the protein environment has been demonstrated, as the isolated cofactor is not active for N_2_ reduction.^25,26^

**Figure 1:**
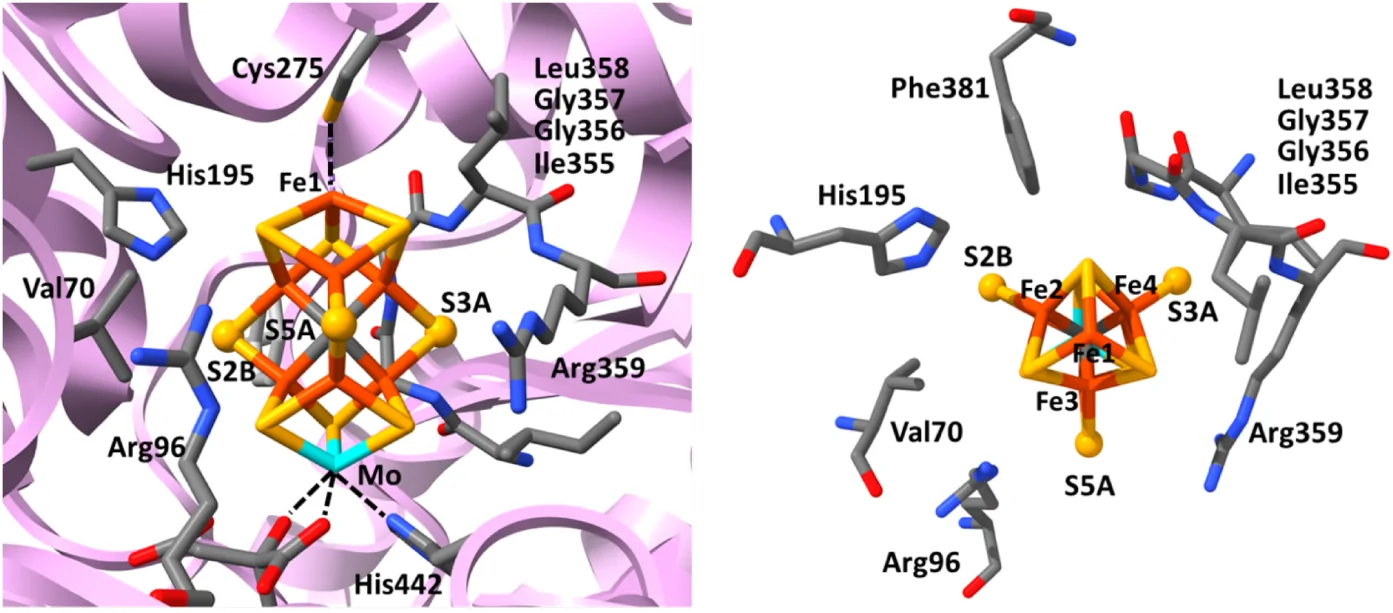
FeMoco-protein interactions within the protein environment (left) and Fe1–Mo top-down view (right) from *A. vinelandii* (PDB ID: 3U7Q). Fe and S atoms and the residues are labeled with respect to the crystallographic notation. Nitrogen atoms are shown in blue, oxygen in red, and carbon in gray. Cofactor ligands Cys275, His442 (residue numbering from *A. vinelandii*), and homocitrate are omitted for clarity, where necessary.

Spectroscopy has been indispensable in elucidating the geometric and electronic structures of catalytically active sites. Among the available techniques, X-ray spectroscopies offer a distinct advantage because they are inherently element-specific, allowing complex multinuclear active sites to be investigated from both the metal and ligand perspectives.^27–29^ The pre-edge region in ligand K-edge XAS provides a direct probe of ligand p character mixed into metal d orbitals, enabling quantitative assessment of metal–ligand covalency in complex metalloclusters.^30,31^ This has been crucial in bioinorganic chemistry, where reactivity and redox behavior often depend on delocalization of electron density across metal–ligand frameworks rather than formal metal oxidation states alone. For FeMoco, direct experimental investigation of individual belt sulfides is challenging because of the presence of multiple atoms, both within the clusters and in the surrounding protein (approximately 142 sulfur atoms in the *A. vinelandii* NifD_2_K_2_ MoFe protein, including 110 protein-derived sulfur atoms and 32 inorganic sulfides).^32^ A strategy to overcome this challenge involves the selective substitution of sulfide ligands with selenide (Se), which is isovalent with sulfur.^33–36^ Previous studies have shown that Se can selectively replace the S2B belt sulfide during turnover under proton-reducing conditions or substitute the S3A and S5A belt sulfides during turnover with N_2_ or C_2_H_2_.^10^ In the latter case, Se occupies the S3A and S5A positions with approximately equal probability, as determined by single-crystal anomalous dispersion measurements (Figure S1). Importantly, the Se-incorporated MoFe retains catalytic activity toward N_2_ reduction.^10^ Subsequent Se K-edge HERFD XAS studies by our group revealed clear spectroscopic differences between these positions, including variations in pre-edge intensity and edge energy.^37^ The pre-edge feature of Se K-edge HERFD XAS directly probes the unoccupied Fe-based orbitals that interact with the Se^2–^ (Se2B bridging Fe2–Fe6; Se3A/5A bridging the Fe4–Fe5/Fe3–Fe7, respectively), and indicates a high degree of electronic asymmetry at the belt positions. Correlating the experimental results with DFT calculations on dimeric complexes, we previously proposed that the Fe2–Fe6 pair is best described as an antiferromagnetically coupled Fe^3+^ pair, while the Fe3/4/5/7 sites exhibit more mixed-valence, Fe^2.5+^, character.^37^ However, the origin of these spectral differences and the extent to which they arise from intrinsic cluster properties versus protein-environment effects remained unclear. In particular, it is not yet understood how the secondary-sphere amino acid residues surrounding the cofactor influence the electronic structure of individual Fe–Se (or Fe–S) bonds. Because these interactions involve weak H-bonds, electrostatic effects, and steric constraints, they are difficult to isolate experimentally. Computational approaches on the full Se-FeMoco, and, more importantly, those that explicitly include the protein environment, are therefore essential for interpreting the spectroscopic data. Secondary-sphere interactions play a central role in tuning the properties of biological metal active sites.^38–41^ Hydrogen-bonding interactions are well-known to modulate reduction potentials, metal–ligand covalency, and proton-coupled electron-transfer processes by influencing the distribution of electron density around the active site.^42–46^ The pre-edge intensity, diagnostic of Fe–S bond covalency, has been shown to be inversely proportional to the reduction potential of Fe–S clusters.^47^ The decrease in Fe–S covalency induced by H-bonding was proposed to preferentially stabilize more reduced Fe-centers.^48^ In Fe–S systems, for example, hydrogen bonding to sulfur ligands decreases metal–ligand covalency and preferentially stabilizes more reduced electronic states.^49–52^ Similar principles have been invoked to rationalize reactivity in nonheme iron enzymes and Cu–S proteins, where local electrostatic interactions can influence substrate activation and bond-formation pathways.^53–58^ In contrast, hydrophobic interactions are often viewed primarily in terms of substrate access or steric control,^59–61^ and their direct influence on electronic structure remains less well understood. Understanding how distinct secondary-sphere interactions shape the electronic structure and reactivity of metallocofactors remains a central challenge in bioinorganic chemistry.

In the present study, we combine QM/MM and TD-DFT calculations of Se K-edge XAS and compare with experimental data to investigate how the protein matrix modulates the electronic structure of Se-substituted FeMoco. Our calculations clearly indicate that it is not a Se-specific observation but also for S. By comparing an isolated cluster with the cluster embedded in the protein environment, we identify the specific interactions responsible for generating electronic asymmetry among the belt sulfides. Furthermore, by selectively removing amino acid residues near each belt position, we evaluate their individual contributions to Fe–Se/S covalency and how the environment surrounding the 2B position promotes localization of electron density onto the belt chalcogenide. In contrast, the 3A/5A belt positions experience interactions that exert a direct electronic influence, not only shaping the steric landscape but also tuning the local electron density and reactivity. Rather than serving merely as structural or steric elements, these residues can actively modulate the electronic environment of FeMoco, influencing local electron density, metal–ligand covalency, and site-selective reactivity. This insight expands the conventional view of secondary-sphere control and underscores that even well-characterized hydrophobic pockets can play a direct, functional role in determining the cofactor’s chemical behavior.^62–64^

## 2 Methods: Computational Details

### 2.1 Setup of QM and QM/MM Calculations

The QM/MM model of FeMoco is based on the *Azotobacter vinelandii* MoFe protein crystal structure (PDB 3U7Q),^5^ that has been previously reported.^65^ The identities of the 2B, 3A, or 5A positions were selectively changed from sulfide to selenide for the selenium-substituted FeMoco structure.

Geometry optimizations within the QM/MM framework were conducted using the ASH program,^66,67^ which integrates OpenMM^68^ for molecular mechanics (MM) and the ORCA electronic structure package (version 5.0.3) for QM calculations.^69,70^ The MM region was parametrized using the CHARMM36 force field,^71^ consistent with prior QM/MM investigations of FeMoco.^12,65,72^ These optimizations employed the geomeTRIC optimizer^73^ utilizing HDLC (hierarchical delocalized internal coordinate) internal coordinates.^74^ Covalent QM/MM boundaries were managed with a link-atom technique complemented by a charge-shift correction scheme^75^ to ensure accurate continuity between QM and MM regions.

All model systems were optimized using the r^2^SCAN density functional^76^ in conjunction with the D4 dispersion correction,^77,78^ that has been benchmarked for iron sulfur compounds.^79^ The cofactor’s Fe, S, Se, and interstitial carbide were treated with the ZORA-def2-TZVP basis set,^80,81^ while Mo was modeled using the all-electron SARC-ZORA-TZVP basis set.^82^ The remaining atoms were described with the ZORA-def2-SVP basis set.^83^ The Split-RI-J approximation was applied using ORCA’s decontracted SARC/J auxiliary basis set.^84^ During optimization, the DefGrid2 integration grid was employed.^85^

The metallocluster was modeled in the E_0_ resting state whose oxidation state is assigned as [MoFe_7_S_8_(Ch)­C]^1–^, where Ch = S or Se. The three carboxylate groups of the *R*-homocitrate were unprotonated, and the metal-coordinating OH group of homocitrate was protonated, giving a total charge of −3. The primary coordination sphere was defined as the residues directly coordinated to FeMoco, which includes Cys275 (α subunit numbering, *A. vinelandii*) and His442. The cofactor and its primary coordination sphere are defined as the QM(1°)/MM model and has 56-atoms and a net charge of −5. The QM model has the same composition, respectively truncating Cys275 and His442 as methylthiolate and 4-methylimidazole (Figure S2). The continuum solvation model (CPCM) with a dielectric constant of ε = 4.0 was used in the QM model.^86^ Unless otherwise stated, ORCA v.5.0.4 was used.^69,70^

The secondary coordination sphere was defined as the residues whose heavy atom separation from the cofactor and its primary coordination sphere is less than the sum of their van der Waals radii^87^ in the 3U7Q structure. This includes Val70, Arg96, Gln191, His195, Tyr229, Arg277, Ser278, Ile355, Gly356, Gly357, Leu358, Arg359, Glu380, and Phe381 (Figure S3A). The QM-region does not include crystallographic water molecules. Only the amino acid backbone, rather than the entire residues, were included for Leu358 and Arg359. The Arg residues were in their cationic state while Cys275 and Glu380 were anionic, all other residues being neutral. The His195 and His442 residues were in their respective ε- and δ-protonation states. The QM region and net charge are respectively 191 atoms and −4. The active region included 990 atoms (Figure S4), an approximately 10 Å radius with respect to the interstitial carbide, their residue identities provided in Section 2 of the Supporting Information.

The cofactor’s electronic structure was treated with broken-symmetry-density functional theory (BS-DFT). The BS-solutions are found by flipping the net-spin of three Fe-centers from α- to β-, converging from the ferromagnetic *M*_s_ (spin projection quantum number) = 35/2 to 3/2 state. There are ten distinct BS-solutions with respect to Noodleman’s original notation^88^ that increases to thirty-five following descents from *C*_3_-symmetry. Our notation assigns the Fe-sites with net β-spin, with respect to the cofactor’s crystallographic labeling. Each structure’s BS −235, −247, and −346 solutions, belonging to Noodleman’s BS7-class, were considered that are reported as the lowest energy for the cofactor’s E_0_ state.^89^ Members of Noodleman’s BS10-class that have net α-spin at the Fe-centers coordinated by a common μ_2_-bridge were also considered (BS10–135 and BS10–147 for Se2B; BS10–125 and BS10–146 for Se5A; BS10–127 and BS10–136 for Se3A). The BS-solution’s identity was assigned with respect to the Hirshfeld spin population of the Fe-sites (Tables S5–S7).^90^ The metal center local oxidation states were assigned with respect to their Pipek-Mezey (PM) localized orbitals (Figure S6).^91^

### 2.2 Computations of X-ray Absorption Spectra

Following geometry optimization within the QM/MM framework, subsequent calculations were performed on the optimized QM region without electrostatic embedding, in order to isolate the electronic structure effects of the QM region itself. The Se and S K-edge X-ray absorption spectra (XAS) were computed using time-dependent density functional theory (TD-DFT) with the r^2^SCAN and TPSSh^92^ functionals (Figure S13) and the basis sets described above and the CPCM (ε = 4.0).^86^ Up to 500 transitions were calculated for each core orbital to capture the spectral rising edge feature. The spectra were generated with the orca_mapspc utility program with a full-width at half-maximum broadening of 2.0 eV for each transition. The calculated Se transition energies were shifted by +71.55 eV, in reference to the deviation between the experimental and calculated spectrum of [Et_4_N]­[SeH].^37,93^ The analogous S K-edge XAS was shifted by +38.86 eV, as referenced to [2Na]­[Se].^94^ The oscillator strength of the transitions were scaled by 0.20.

The σ- or π-bonding character of the pre-edge transitions of α- and β-spin were assigned by the acceptor orbital of the natural transition orbitals (NTOs),^95^ as shown in Figures S14–S16. The total Se or S p-orbital contribution to the NTOs was calculated by a Hirshfeld population partitioning scheme using Multiwfn v3.8.^96,97^ This value corresponds to the covalency (α^2^) of the S- or Se–Fe bonding interaction. The oscillator strength of each transition (Int­[1s → ψ*]) was the sum of the electric dipole, electric quadrupole, and magnetic dipole transitions. The 3 times of the ratio of the Int­[1s → ψ*] and respective α^2^ is defined as the dipole integral (*I*(S) or *I*(Se)), whose values are included in Tables S9–S13 for each model’s pre-edge transitions.

### 2.3 Characterization of Noncovalent Interactions

To characterize the secondary coordination sphere interactions, an energy decomposition analysis employing the extended transition state method combined with natural orbitals for chemical valence (ETS-NOCV) was performed using Multiwfn (version 3.8).^96,98^ The TPSSh hybrid functional and the computational procedure detailed above was used. The two-fragment scheme were defined as a select residue (Fragment 1) and the remaining atoms of the QM(2°) model (Fragment 2). The BS-solution of Fragment 2 was kept consistent with the total system (Fragment 1, 2). The CPCM model was used for each component of the fragment analysis and does not include the MM region as point charges. The isolated fragments are not individually optimized, and their coordinates were fixed to those of the total system. The total charge of each fragment was defined consistently with the total system, with Arg assigned a positive charge, Glu assigned a negative charge, and the remaining residues assigned neutral charges. The NOCV pair densities, together with their associated orbital interaction energies and eigenvalues, are shown in Figure S20 for Se-FeMoco and Figure S21 for WT.

### 2.4 E_0_ Protonation Calculations

Geometry optimizations within the QM/MM framework were conducted keeping all parameters similar to our QM(2°)/MM model. Herein, a proton was introduced into the system, changing the overall charge of the system to −3. The BS7–346 solution was applied for protonated S2B, BS7–247 for protonated S5A, and BS7–235 for protonated S3A. The respective BS solutions were found by flipping the net spin of three Fe centers from α to β, converging from the ferromagnetic *M*_s_ = 35/2 to the *M*_s_ = 3/2 state. Each protonated structure was independently optimized using both the r^2^SCAN and TPSSh functionals shown in Table S14. The relative electronic energies (ΔE) of the QM(2°)/MM optimized structures do not include ZPE or thermal corrections that could affect quantitative trends.

## 3 Results and Discussion

The three μ_2_-belt sulfides of FeMoco are often drawn as formally equivalent structural elements of the cofactor. However, the experimental Se K-edge HERFD XAS results and the known reactivity of the S2B position already argue against a purely symmetric description of the belt region.^12,37^ In particular, the enhanced pre-edge intensity observed for Se at the 2B site (Figure 3A) relative to Se3A/5A suggests that the Fe–Se interactions at these positions are electronically distinct. A useful starting point is the asymmetric distribution of amino acid residues around the three belt sites (Figure 1). As discussed earlier, His195, Phe381, and Val70 are located near the S2B position, Arg96 and Arg359 are proximal to S5A, whereas the S3A position is primarily influenced by the peptide backbone amides of Ile355, Gly356, Gly357, and Leu358 (Figure S3B). In other words, the three belt positions are not embedded in equivalent electrostatic or H-bonding environments, even though the isolated cofactor is *C*_3_ symmetric.^14,15^ H-bonding residues such as His195 ^17–21^ and Arg96 ^24^ have been shown to influence proton delivery pathways and substrate reduction behavior, consistent with their role in shaping the local electrostatic environment. At the same time, mutations of Val70,^22,23^ referred to as nitrogenase’s “gatekeeper”, which are positioned near the S2B site and are not involved in H-bonding, have been reported to impact substrate access, diffusion pathways, and overall reactivity at the active site.^23,25^ The central objective of this work is therefore not simply to reproduce the spectral differences, but to understand their origin and, more importantly, to determine how the surrounding protein matrix generates the electronic asymmetry that ultimately gives rise to selective reactivity at the belt positions.

### 3.1 Structural Asymmetry Emerges Only When the Cofactor Is Embedded in the Protein Environment

To examine whether the experimentally observed belt chalcogenide differentiation is already encoded in the inorganic core itself, we first compared three levels of modeling: the isolated cofactor (QM, Figure 2A), the cofactor embedded in the protein matrix (MM region) but without explicit secondary-sphere residues in the QM region (QM(1°)/MM, Figure 2B), and the cofactor with both the protein environment and a larger QM region that includes the key nearby residues (QM(2°)/MM, Figure 2C).

**Figure 2:**
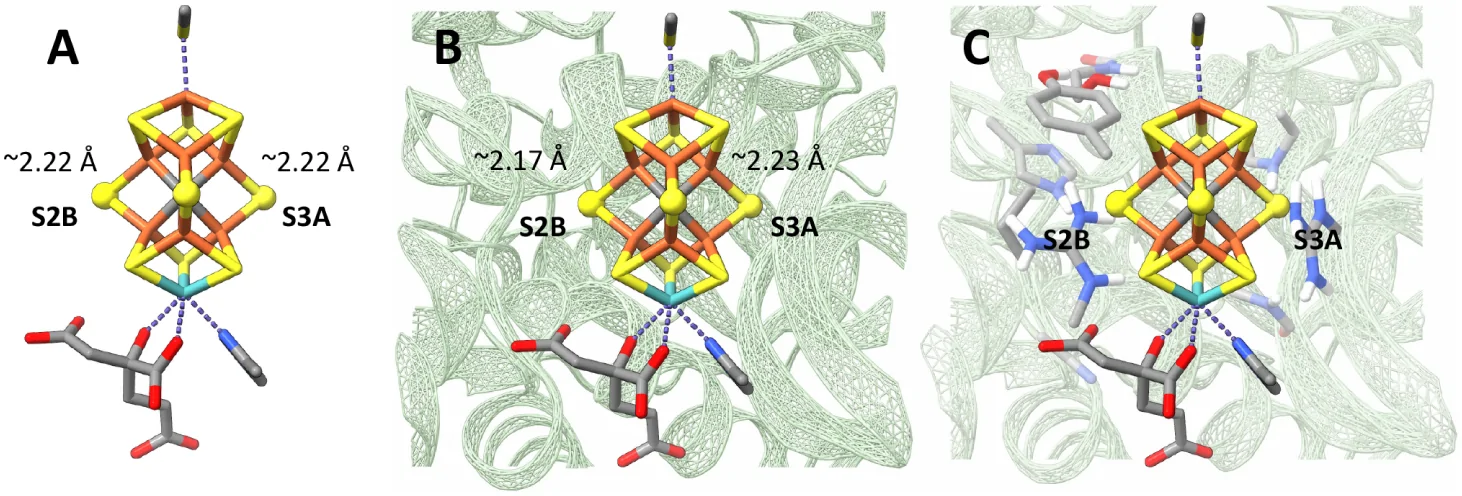
Three model structures consist of: (A) the isolated cofactor (**QM**) with 56 atoms in the QM region, (B) the cofactor with 56 atoms in the QM region embedded in the protein matrix (MM region shown as green ribbon treated as point charges) **[QM­(1°)/MM]**, and (C) the cofactor with 191 atoms in the QM region including the key nearby residues embedded in the protein matrix **[QM­(2°)/MM]**. The models are shown with respect to the WT-cofactor, hydrogen atoms being omitted for clarity. The average μ_2_S–Fe distances are listed for S2B and S3A/5A (Tables S2–S4).

Within a broken symmetry (BS) framework, the cofactor’s resting state is best described by the BS7 class of solutions, with respect to notation introduced by Noodleman.^89^ The BS7 class assigns Fe-sites bridged by a common belt sulfide to be antiferromagnetically coupled, which has three distinct solutions when accounting for the cofactor’s descent from *C*_3_-symmetry. While the three BS-solutions belonging to the BS7 class have near degenerate energies, the Fe-sites possess distinct oxidation states in which the belt sulfide coordinates an Fe^3+^ or mixed-valence Fe^2.5+^ site (Figure S6). By selecting specific BS7-solutions, we can therefore isolate the influence of the Fe-site oxidation states on the XAS spectrum of the coordinating bridging ligand. We have previously detailed that more oxidized Fe^3+^ centers should exhibit more covalent Fe–Se or Fe–S bonding interactions than the more reduced Fe^2.5+^ sites.^37^ To maintain a consistent local oxidation state assignment at the Se-substituted site across all three structures, we selected the spin isomer in which the Fe neighboring the μ_2_-selenide is in the +3 state. This resulted in BS7–346 for Se2B, BS7–247 for Se5A, and BS7–235 for Se3A. We also consider the second lowest-lying solutions, which belong to the BS10 class and feature ferromagnetically coupled Fe-sites coordinated to the belt sulfide. These BS-solutions allow us to isolate the impact of magnetic coupling on the pre-edge spectrum of the bridging ligand.^99^ The corresponding solutions are BS10–135 and −147 for Se2B, BS10–125 and −146 for Se5A, and BS10–127 and −136 for Se3A. We report that their relative energies are conserved in the wild-type (WT)- and selenide-substituted FeMoco that suggests that selenide does not change the cofactor’s underlying electronic structures (Table S1).

All calculations start from a single crystallographic geometry. We note that full ensemble sampling is beyond the scope of this study. To assess how representative this geometry is, we compared the WT structure used in our model (PDB ID 3U7Q, 1.0 Å)^5^ with the independently determined Se-substituted structure (PDB ID 5BVG, 1.6 Å).^10^ The key active-site residues and cofactor-coordinating side chains superimpose closely (RMSD = 0.093 Å; Figure S5), and the hydrogen-bonding partners included in the QM(2°)/MM model retain the same positions and connectivity.

The average and individual Se–Fe bonds are collected in Table 1 and Tables S2–S4, respectively, for our three computational models. The isolated QM model shows essentially equivalent average Se–Fe distances for Se2B and Se3A/5A. By contrast, upon embedding the cofactor in the protein environment, the 2B position becomes structurally distinct: the average Se–Fe distance at Se2B shortens by approximately 0.05 Å relative to Se3A/5A, and the same trend is reproduced for the WT S2B versus S3A/5A distances. The longer Se–Fe bond lengths compared to the WT-structure is consistent with the larger radii and was similarly reported in analogous Fe-complexes.^33^

**Table 1:** Average S/Se–Fe Distance (Å)

Position	Exp (Cryst)	Exp (EXAFS)	QM	QM(1°)/MM	QM(2°)/MM
S2B	2.19 ± 0.02^a^	-	2.22 ± 0.07	2.17 ± 0.03	2.17 ± 0.03
S3A/5A	2.23 ± 0.02^a^	-	2.22 ± 0.01	2.23 ± 0.02	2.22 ± 0.02
Se2B	2.30 ± 0.04^b^	2.29 ± 0.02^d^	2.35 ± 0.02	2.31 ± 0.05	2.30 ± 0.04
Se3A/5A	2.31 ± 0.03^c^	2.39 ± 0.02^d^	2.36 ± 0.02	2.37 ± 0.03	2.35 ± 0.03

a PDB ID (structure resolution): 3U7Q (1.0 Å)^5^.

b PDB ID (structure resolution): 5BVG (1.6 Å)^10^.

c PDB ID (structure resolution): 5BVH (1.53 Å)^10^.

d Reported from experimental HERFD Se K-edge EXAFS.^37^ The bond lengths of the Se3A and Se5A sites are presented as an average. The calculated bond lengths are with respect to the optimized geometries of their Ms = 3/2 BS–235 (S2B), BS–235 (S3A/5A), BS–235 (Se2B), BS–247 (Se5A), and BS–346 (Se3A) solutions.

These results are in good agreement with the experimental crystallographic and Se K-edge HERFD EXAFS trends and show that the distinction of the 2B site is not an inherent property of the bare cluster, but emerges only when the cluster is placed in its proper protein context. The analogous Fe–S bond lengths for the WT-protein are not accessible by HERFD K-edge XAS because the μ_2_-sulfides are not chemically distinct from the other sulfides and Cys residues.

We observe minor variations in the S- and Se–Fe distances when expanding the QM-region to include the cofactor’s secondary coordination sphere in the QM(2°)/MM model (Table 1). This demonstrates that the belt-positions distinct geometric structures are dominantly an electrostatic effect of the surrounding protein environment. The contracted bond lengths of the 2B, compared to the 3A/5A sites, is consistent with greater covalency to the Fe-sites which is consistent with our reported spectroscopic trends detailed below.

### 3.2 Reproducing the Se HERFD XAS Trends Requires Explicit Inclusion of the Protein Environment

TD-DFT calculations of the Se K-edge XAS spectra were carried out at each Se-substituted belt site. The experimental HERFD data, as discussed earlier, show a clear increase in pre-edge intensity and a shift in the rising-edge to higher energy for Se2B relative to Se3A/5A (Figure 3A). We note that our calculated K-edge XAS spectra in Figure 3B–3D focus on the pre-edge and low-lying rising-edge features because TD-DFT does not adequately describe the nonlocal excitations and multielectron processes that contribute to the higher-energy white-line and continuum region.^100–103^

**Figure 3:**
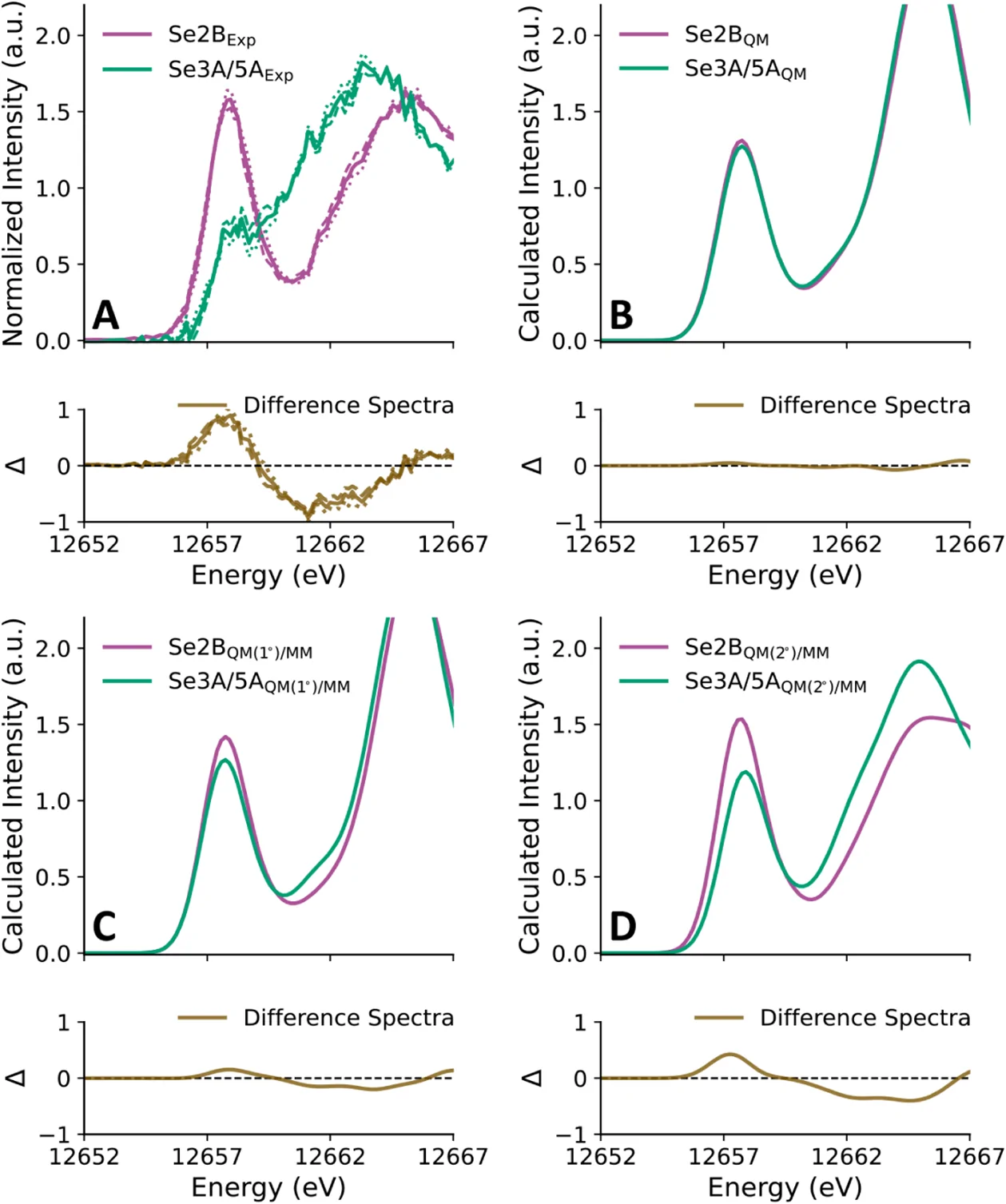
(A) Deconvoluted experimental Se HERFD K-edge XAS spectra for Se incorporated FeMoco, contrasting Se at the μ_2_-belt sulfide positions Se2B (pink) and Se3A/5A (green), adapted from *J. Am. Chem. Soc.* **2019**, *141*, 13676. (B–D) TD-DFT calculated Se K-edge XAS spectra for the three models: (B) **QM**, (C) **QM­(1**°**)/MM**, and (D) **QM­(2**°**)/MM**). The difference spectra of Se2B with respect to Se3A/5A are plotted in brown for each panel. Note, the *Y*-axis range has been intentionally cut off for representation purposes. As one cannot use calculations to predict changes in the white line, our focus was to be consistent in representation with experiments.

In the isolated QM model, the calculated spectra show negligible variations between the belt positions (Figure 3B). These results indicate that the intrinsic electronic structure of the inorganic core alone does not give rise to the pronounced spectral asymmetry observed experimentally. We report negligible variations in the calculated selenide properties of the low-lying BS-solutions, where the coordinated Fe-site has a more reduced mixed-valence Fe^2.5+^ state, suggesting the inequivalence between the three belt positions is not attributable to the local Fe-oxidation state (Figures S7–S10). The analogous calculated spectra for the BS10 solutions show a substantial decrease in pre-edge intensity (Figure S11), consistent with the smaller number of holes with Se character available for the transitions. This demonstrates that local ligand-based electronic properties can provide insight into the overall magnetic interactions within the cofactor.

The QM(1°)/MM model captures the impact of the cofactor’s geometric asymmetry, resulting from the electrostatic modeling of the protein environment in the cofactor’s optimization. The calculated QM(1°)/MM spectra show a moderate increase in the pre-edge intensity of 2B, which is consistent with the 0.05 Å bond contraction reported in Table 1 (Figure 3C). We quantify the distinction of the 2B and 5A/3A sites using the relative areas of their total pre-edge features (Table S8).

Including the electrostatic protein environment as point charges (Figure S12) increases the pre-edge area difference from 9.0%, produced by the geometric asymmetry alone, to 24.5%. This is comparable to the 21.9% difference obtained upon explicit inclusion of the secondary-sphere residues in the QM(2°)/MM model (Table S8), indicating that the influence of the secondary coordination sphere on the pre-edge intensity is predominantly electrostatic in origin. The explicit QM treatment additionally provides a molecular description of the interactions responsible for this perturbation. The calculated pre-edge and rising-edge shift for Se2B (Figure 3D) reproduce the experimentally observed differences. These results demonstrate that the protein environment plays a crucial role in generating the spectroscopic asymmetry between belt sulfides.

To evaluate whether this behavior is specific to Se substitution or reflects a more general property of the native cofactor, analogous calculations were performed for the S K-edge spectra of the WT FeMoco. Notably, the calculated S K-edge spectra show the same qualitative trend (Figure 4), with the S2B position exhibiting a higher pre-edge intensity relative to the S3A and S5A positions. The WT and Se-substituted cofactors exhibit 19.6 and 21.9% differences, respectively, in the pre-edge area between the 2B and 3A/5A sites. The smaller distinction in the WT-system is consistent with the smaller ionic radius of S with respect to Se, as discussed in the following section. This demonstrates that the electronic asymmetry between the belt positions is an inherent feature of the cofactor in its native protein environment and is not introduced by Se substitution. Taken together, these results show that Se substitution does not artificially induce electronic differentiation within the cofactor, but rather provides a spectroscopic handle to experimentally resolve an intrinsic asymmetry that is already present in the WT system in the resting state. The Se K-edge HERFD XAS measurements, therefore, directly report on the underlying electronic structure of the belt sulfides, enabling a site-specific understanding of how the protein environment governs their reactivity.

**Figure 4:**
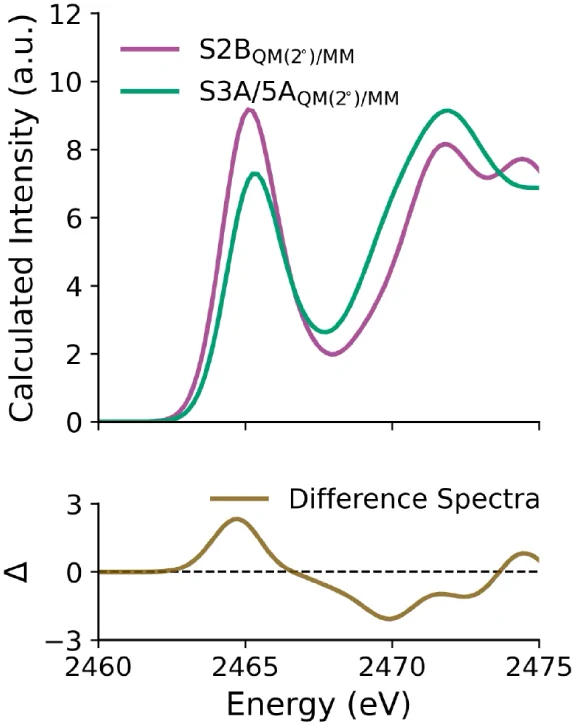
TD-DFT calculated S K-edge XAS spectra for the **QM­(2**°**)/MM** model. Difference spectra (S2B–S3A/5A) are plotted in brown to illustrate site-specific spectral distinctions.

The site-dependent spectral differences are observed experimentally in solution, where conformational heterogeneity is inherently sampled. Their persistence in the experimental spectra suggests that the underlying electronic structure effects are not restricted to a single crystallographic conformation. The calculations reproduce the same overall trends and provide a framework for understanding their electronic origin. Ensemble sampling is a natural next step for quantifying how protein conformational dynamics tune these effects.

### 3.3 Origin of the Pre-Edge Intensity Differences: Covalency and Dipole Integral Contributions

The pre-edge transition in Se K-edge XAS results from the excitation of an electron from the Se 1s core orbital to the antibonding orbitals resulting from the Se–Fe interactions. The observed intensity can be related directly to the calculated oscillator strength (eq 1), which is 1/3 of the product of the covalency (*α* ^2^, defined by the ligand’s p character in the acceptor orbital) and the transition dipole integral, *I*(Se), for its localized 1s to *n*p transition (eq 2).^104^ In this regard, it is pedagogical to compare the intrinsic Se 1s to 4p dipole integrals to those of a Se 1s to 5p transition. As shown in Figure 5A, the more compact 4p orbitals have a steeper slope when one plots oscillator strength against the percentage of Se *n*p character, indicating that the intrinsic *I*(Se_4p_) dipole integral is higher relative to (Se_5p_), where the acceptor orbitals are more diffuse. While this highlights an extreme case at the atomic limit, it follows that the nature of the metal–ligand bonding interaction will similarly modulate the dipole integral, as highlighted in Figure 5B–D.

1 $$\mathrm{Int}\left[1s\to \psi^{*}\right]=\frac{1}{3}\alpha^{2}\cdot I\left(\mathrm{Se}\right)$$

$$I\left(\mathrm{Se}\right)={\left|\left\langle {\mathrm{Se}}_{1s}|r|{\mathrm{Se}}_{np}\right\rangle \right|}^{2}$$ 2

**Figure 5:**
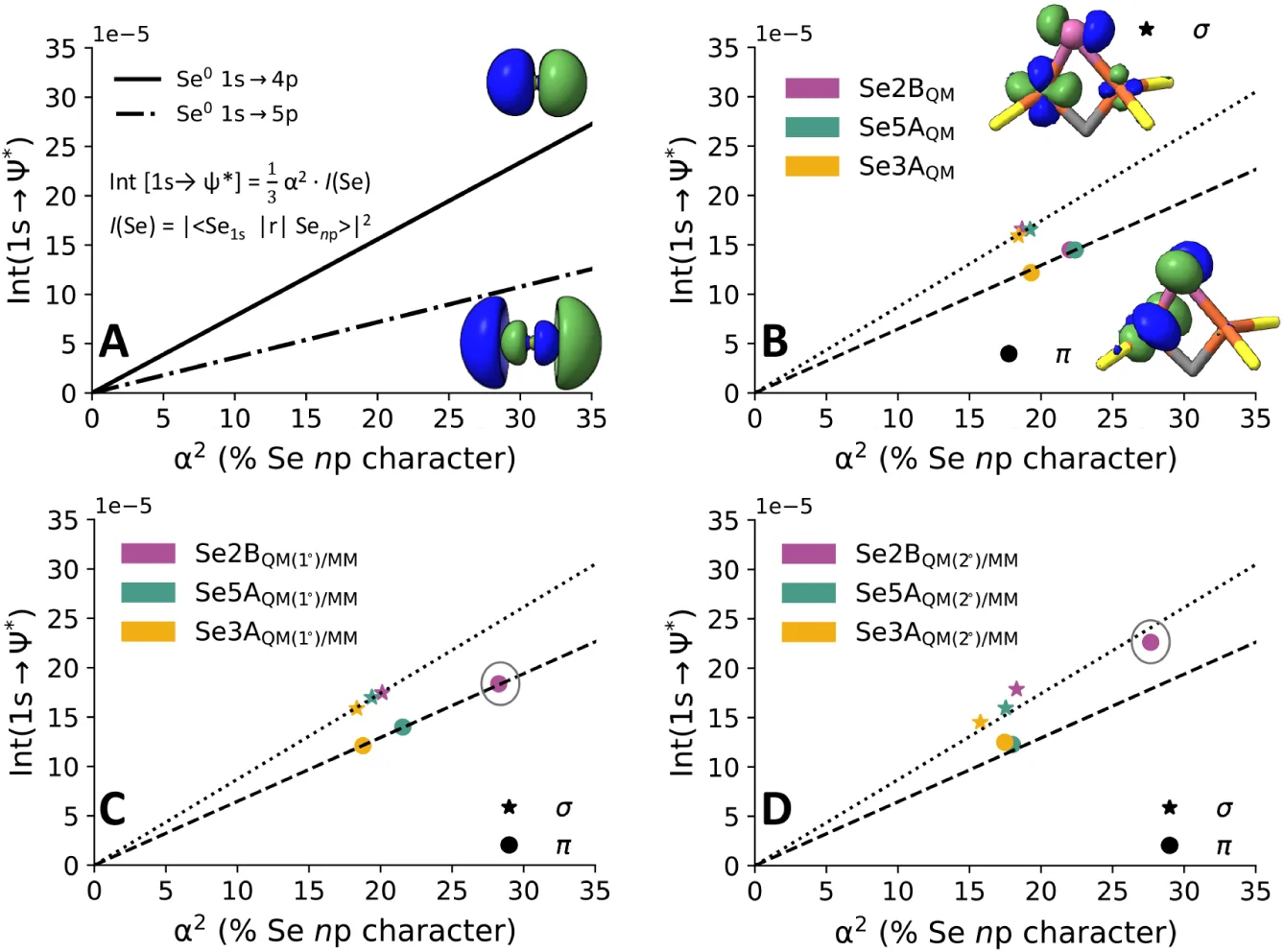
(A) TD-DFT-calculated oscillator strengths (Int­(1s → ψ*)) plotted against the percentage of Se 4p character (solid line) and Se 5p character (dashed-dotted line) for Se^0^. Here α^2^ represents the Se *n*p character, and the slopes are set by the calculated atomic 1s-4p and 1s-5p transition dipole integrals. The *x*-axis is limited to 35% to facilitate comparison with the molecular σ- and π-type acceptor orbitals in panels B–D. (B–D) (Int­(1s → ψ*)) vs % Se *n*p character (α^2^) per hole for both σ (star markers) and π (circle markers) pre-edge transitions in the (B) QM, (C) QM(1°)/MM and (D) QM(2°)/MM models, with Se positioned at 2B (pink), 5A (green) and 3A (deep yellow). Linear trendlines (dotted, σ; dashed, π) were fit to the QM model in (B) and held fixed for direct comparison in (C) and (D). The gray circle is to guide the readers for the Se2B π-bonding properties.

In the present study, the intensity and covalency of the pre-edge transitions were obtained from the TD-DFT calculated spectra, the slope being proportional to the dipole integral. The resulting relationships are shown in Figure 5B–5D for the individual Se2B, Se5A, and Se3A sites in our three computational models. The pre-edge includes transitions of σ- or π-antibonding character, with respect to the Se–Fe coordination (Figure 5B). As anticipated, the dipole integrals are distinct for the transitions into the antibonding Se–Fe σ- and π-orbitals. This is consistent with the greater number of nodes in π-interacting orbitals, and the fact that π antibonding orbitals are more dispersed from the 1s core, resulting in a reduced dipole integral.^105^ We note that the analogous characterization of the WT-cofactor is complicated by the delocalization of the pre-edge NTO’s across multiple S-centers, which prevents the assignment of covalency values to discrete belt positions (Figure S17). This limitation further highlights the utility of selective Se-substitution for characterizing FeMoco’s electronic structure.

We show the changes in the intensities, α^2^, and *I*(Se) among our three computational models in Figure 6. The relative perturbations of these spectroscopic terms for the QM(1°)/MM relative to the QM models in Figure 6A, capturing the impact of the distinct Se–Fe bond distances described in Table 1. Dipole integral values were extracted individually for each Se site using the eq 1. Figure 6A shows the impact of the distinct cofactor geometries between the QM(1°)/MM and QM models that are collected in Table 1. We highlight that the largest perturbation to the QM(1°)/MM pre-edge manifold is the +26.5% increase in the π-transition of the Se2B site, noting that this Se-π orbital is the lone pair that is protonated during reactivity. The σ-transition of the Se2B site only increases by +5.2%, which suggests it is less susceptible to changes from the surrounding protein environment. Model nonheme Fe complexes have previously demonstrated the heightened sensitivity of Fe–S π-bond covalency to changes in the secondary coordination sphere.^50,106^ Both the σ- and π-transitions of the Se3A/5A sites are minimally impacted in the QM(1°)/MM model, which agrees with the conserved Se–Fe bond lengths between the models (Table 1). We attribute the dominant intensity increase at the Se2B site to the 0.05 Å contraction of the Se2B–Fe bond length in the QM(1°)/MM model, which should increase the Se–Fe bonding interaction. We report minor fluctuations in the dipole integrals, so that the slopes as illustrated in Figure 5B–C are conserved between the QM and QM(1°)/MM models.

**Figure 6:**
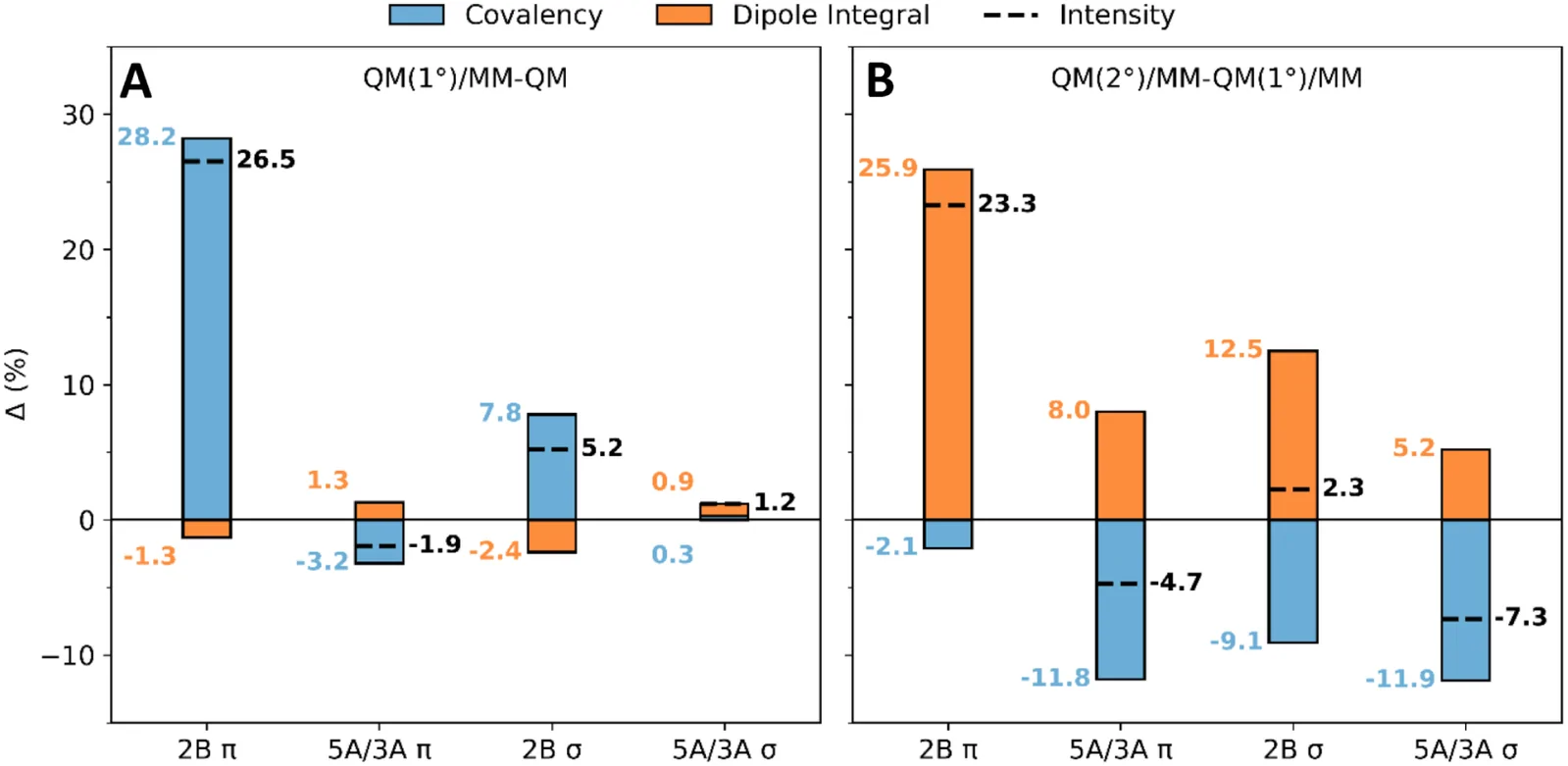
Relative change in the Se K-edge XAS pre-edge intensity (Δ) of the QM(1°)/MM with respect to the QM model (A) and QM(2°)/MM with respect to the QM(1°)/MM model (B). The covalency (blue) and dipole integral (orange) contributions to the total intensity (dashed black line) is provided for the σ and π transitions of the respective 2B and 3A/5A Se-substituted cofactors. The relative change of the QM(1°)/MM and QM(2°)/MM models shows the respective impacts of the cofactor’s geometry and the surrounding residues on the spectrum’s pre-edge. Dipole integral values were extracted individually for each Se site using the eq 1.

Figure 6B shows the perturbation in the spectroscopic values of the QM(2°)­MM model relative to the QM(1°)/MM model, isolating the impact of the proximal amino acid residues on the Se pre-edge. Similar to Figure 6A, the largest change in the pre-edge intensity occurs for the Se2B π-transition, which increases by +23.3%. This change is principally attributed to the +25.9% enhancement in the *I*(Se), consistent with the deviation of the 2B π-transition from the intensity-covalency relationship shown in Figure 5D. The other pre-edge transitions exhibit +5.2 to 12.5% increases in *I*(Se), suggesting the protein environment generally enhances the dipole integral. These changes are partially offset by corresponding −9.1 to −11.9% decreases in covalency, resulting in only minor changes to the net intensities relative to the pronounced effect observed for the Se2B π-transition.

Taken together, these results show that while covalency provides an important baseline for understanding the Fe–Se interactions, perturbations in the dipole transition integral strongly contribute to the differentiation of the belt positions, which is highly sensitive to the local environment. In order to understand the origin of these differences upon inclusion of the secondary-sphere residues, we next examine the role of individual amino acid residues surrounding the belt positions.

### 3.4 Role of Secondary-Sphere Residues in Modulating Electronic Structure

Our QM(2°)/MM model includes the secondary sphere residues in the QM region (Figure 2C). Despite negligible fluctuations in the Se2B–Fe bond lengths, the pre-edge intensity further increases by 29.0% in comparison to the QM(1°)/MM, indicating that the bridging site’s noncovalent interactions with the proximal residues impact the local electronic structure. Notably, this effect is most pronounced in the π-type transitions, where the orientation and spatial extent of the orbitals are particularly sensitive to the surrounding environment. We calculated the Se K-edge XAS spectrum for the Se2B site upon removing the H-bonding (His195) or hydrophobic (Val70 and Phe381) residues from the system, which is shown in Figure 7A and Figure S18. To probe the electronic and electrostatic contributions of Val70 and Phe381 to the Se site, each residue was individually removed from the optimized QM(2°)/MM structure without further geometry optimization, followed by a single-point TD-DFT XAS calculation. By keeping the remainder of the structure fixed, this approach isolates the effect of the respective residue without introducing additional changes due to structural relaxation. These calculations are intended as controlled computational perturbations and not as models of physical mutations, for which relaxation of the surrounding protein environment would need to be considered. We similarly studied the impact of the H-bonding (Arg96 and AA) residues on the Se3A/5A π-transitions. We find that the largest perturbation to the XAS intensity comes from the presence of the hydrophobic Val70 and Phe381 residues, increasing (QM(2°)/MM – QM(2°)/MM without the indicated residue) the intensity by +17.2%. Surprisingly, this change principally derives from the dipole moment increasing by +19.3%, which means the selenide’s π-lone pair becomes more localized on the Se core when interacting with the hydrophobic groups.

**Figure 7:**
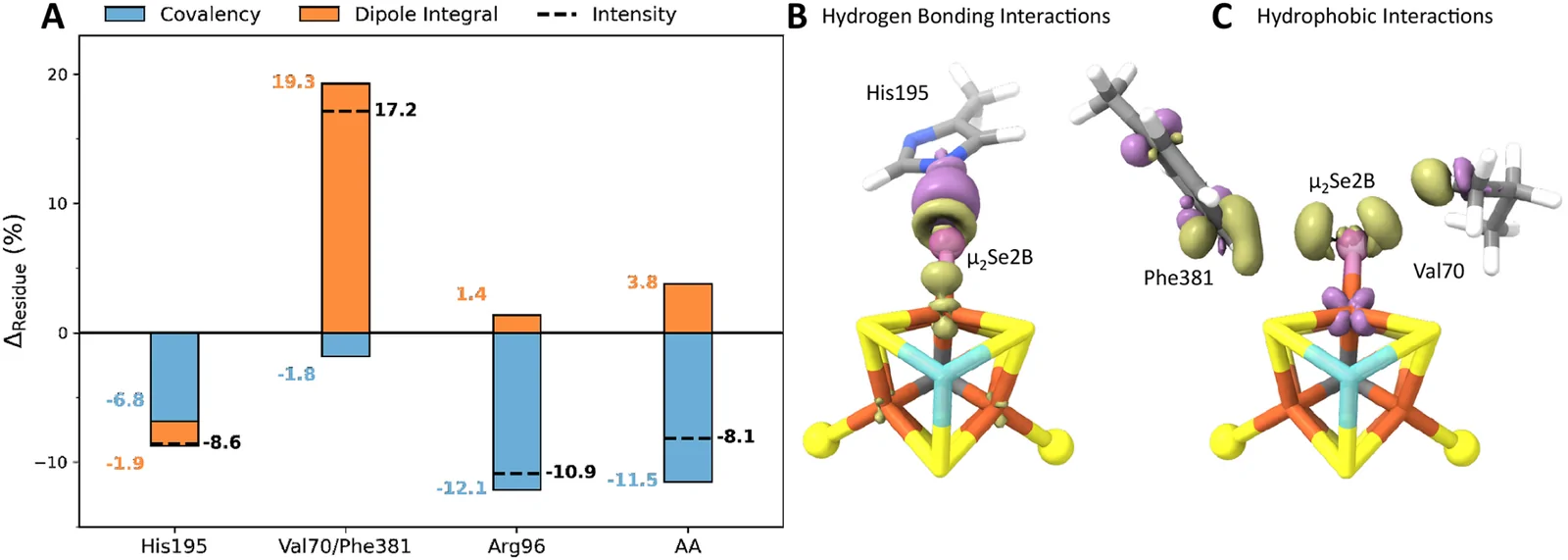
(A) The impact of the titled residues (*x*-axis) on the pre-edge intensity of the π-intensity of the Se K-edge XAS with respect to the QM(2°)/MM model (QM(2°)/MM – QM(2°)/MM without the indicated residue). The covalency (blue) and dipole integral (orange) contributions to the total change (dashed black line) is provided. The Val70/Phe381 and His195 residues are with respect to the Se2B and the Arg96 and AA residues are with respect to the Se3A/5A substitutions. (B–C) The NOCV electron pair densities for the hydrogen bonding His195 (B) and hydrophobic Val70/Phe381 (C) residues on the Se2B (Shown in pink) substituted cofactor. Their noncovalent interactions, resulting in an increase and decrease in electron density are shown in purple and olive, respectively. For clarity, only the [MoFe_7_S_8_SeC] cluster and labeled residues are included.

We report that the H-bonding of His195 to Se2B decreases the π-transition intensity by −8.6%, which is principally attributed to covalency, α^2^ decreasing by −6.8%. We report a similar relationship to the π-transitions at the 3A/5A positions because of their H-bonding interactions. This is consistent with previous reports that H-bonding weakens π-covalency in Fe–S systems.^50^ We also examined the residues surrounding the Se2B site, which exhibit a slight modulation of the pre-edge feature. Their impact is shown in Figure S19 and Table S13, respectively.

Importantly, the combined analysis of covalency and dipole contributions reveals that while H-bonding interactions tend to reduce covalency, the hydrophobic interactions act to enhance the dipole contribution at the 2B position. As a result, the net effect of these competing interactions is a distinct electronic environment at the 2B site that is not present at either the 3A or 5A positions.

Figure 7B, and 7C show how hydrophilic His195 and the hydrophobic Val70 and Phe381 residues perturb electron density in the 2B-substituted cofactor. NOCV electron pair densities highlight regions of electron accumulation (purple) and depletion (olive) associated with these noncovalent interactions. His195 polarizes electron density away from the 2B center toward the H-bonding interaction with the Lewis acidic N–H group, consistent with the −6.8% decrease in covalency shown in Figure 7A. Additional NOCV pair densities for Se-FeMoco are given in Figure S20, and a similar polarization pattern is found for WT-FeMoco (Figure S21). Although Solomon and coworkers previously proposed that hydrogen bonding reduces metal–ligand covalency through localization of electron density on the ligand, Figure 7B indicates that electron density is instead delocalized from the ligand toward the hydrogen bond donor.^50,106^

Comparatively, the Val70 and Phe381 residues polarize the Se-electron density away from the hydrophobic interactions, which enhances electron density in the π-bonding orbitals of the coordinated Fe-sites. Notably, the apparent decrease in Se orbital population at the 2B position is consistent with the 19.3% increase in the dipole integral because the two quantities probe different properties. The Se p character describes the magnitude of the Se contribution to the acceptor orbital, whereas the dipole integral is also sensitive to its radial distribution. Thus, the dipole integral can increase even as the Se p character decreases if the Se p component of the acceptor orbital becomes more radially contracted. Similarly, Hadt et al. showed that repulsive secondary-sphere interactions increase the pre-edge intensity in Cu–S systems, an effect that was attributed exclusively to changes in covalency.^57^ It is worth noting that the influence of noncovalent interactions on FeS clusters has recently been explored outside protein systems.^107^ Bailey reported an analogous effect of proximal aromatic and alkyl groups on Fe–S covalency in host–guest complexes using X-ray photoelectron spectroscopy (XPS).^62,63^

### 3.5 Electronic Asymmetry, Charge Localization, and Implications for Basicity

Studies of ferredoxin models have demonstrated that the cluster p*K*_a_ decreases by approximately one per amide-thiolate hydrogen bond, leading to suppression of thiolate exchange reactions.^108^ Likewise, hydrogen bonding to the thiolate that undergoes protonation in the rate-limiting step inhibits reactivity in [FeFe] hydrogenase mimics.^109^ In contrast, analogous relationships have not been established for steric interactions, even though bulky substituents are routinely employed to stabilize reactive metallocofactor mimics.^110,111^

Similarly, in FeMoco the consequence of these secondary-sphere interactions is a pronounced electronic asymmetry among the belt positions, which is reflected not only in the spectroscopic signatures but also in the underlying electron density distribution. In particular, the presence of hydrophobic residues around the 2B position leads to a localization of electron density at the belt chalcogenide, whereas the other belt positions, influenced more strongly by H-bonding interactions, exhibit a more delocalized electronic distribution.

This difference in electron density localization has direct implications for the effective basicity of the belt sulfides. A more localized electron density at the 2B position corresponds to a higher basicity relative to the 3A and 5A positions, which are stabilized by H-bonding interactions that delocalize electron density. As a result, upon introduction of a proton during the catalytic cycle, the 2B position is preferentially protonated under physiological pH conditions (as the crystal structure utilized was collected at physiological pH 7.5).^5,12^ This basicity trend is illustrated by the relative electronic energies (ΔE) of the QM(2°)/MM optimized structures of the six structural isomers of WT-FeMoco’s E_0_ state with a protonated belt sulfide as shown in Figure 8. While protonation of E_0_ does not by itself establish the preferred protonation site following reduction to E_1_, this result is similar to our previous calculations of the E_1_ state,^12^ which likewise identified S2B as the preferred protonation site. We report an analogous trend for the QM(2°)/MM optimized structures with the r^2^SCAN (Figure 8) and TPSSh (Table S14) DFT functionals.

**Figure 8:**
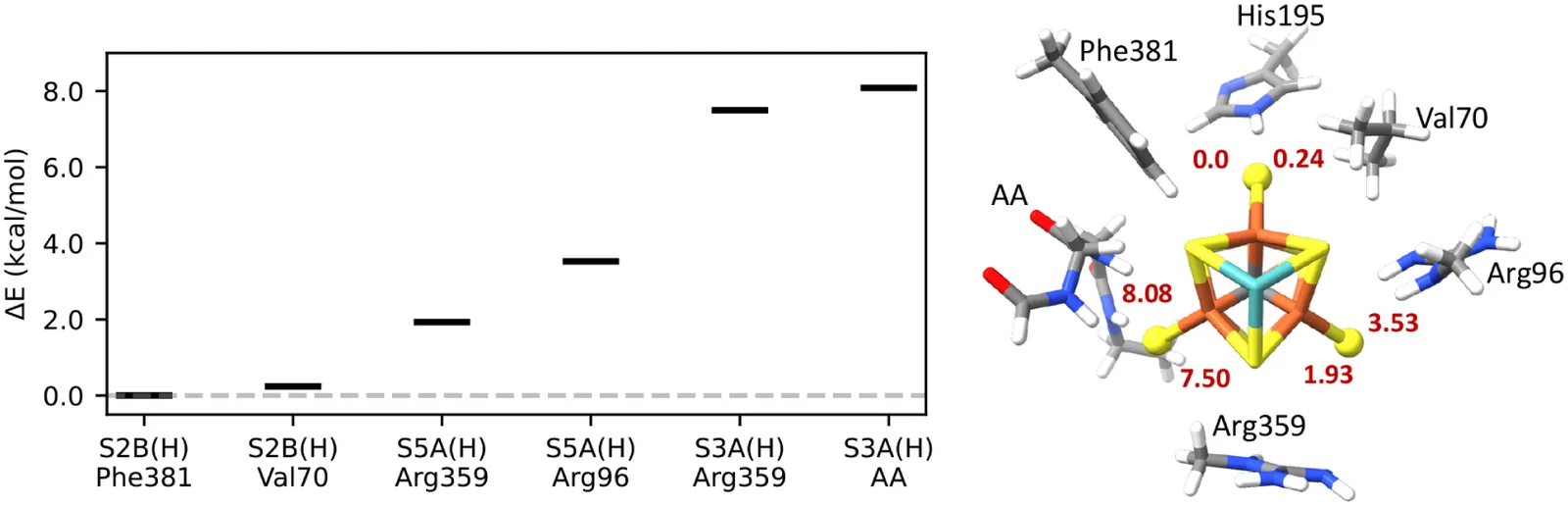
r^2^SCAN calculated relative energies for the μ_2_-sulfide protonated isomers in FeMoco’s resting state (left), also provided in red (right figure), in kcal mol^–1^. The direction of the S–H bond is specified toward an adjacent residue and is mentioned on the *x*-axis (left figure). The BS7–346 solution was applied for protonated S2B, BS7–247 for protonated S5A, and BS7–235 for protonated S3A.

These results therefore provide a direct electronic explanation for the site-selective reactivity of the belt sulfides. The protein environment does not merely modulate the cofactor structure, but actively controls the distribution of electron density through a balance of H-bonding and hydrophobic interactions. In doing so, it establishes a hierarchy of basicity among the belt positions, with the 2B site being uniquely positioned to participate in early protonation steps of the nitrogenase catalytic cycle.

We note that, in the QM(2°)/MM model, the Se2B center has a Hirshfeld charge of −0.21 while the Se5A and Se3A sites have charges of −0.38 and −0.33, respectively. From a simple electrostatic perspective, these charges would suggest preferential protonation at the more negatively charged 5A or 3A sites, contrary to the calculated preference for protonation at 2B. This apparent discrepancy highlights that net atomic charge alone is not a sufficient descriptor of site-specific basicity. Hirshfeld charges reflect the total electron population assigned to the Se center and are strongly influenced by Fe–Se covalency. As the Fe–Se interaction becomes more covalent, a greater fraction of the Se electron density is shared with the coordinated Fe centers, resulting in a less negative atomic charge. The Hirshfeld charge also does not describe how the Se-based electron density is spatially distributed among individual orbitals. The dipole integral provides complementary information because it is sensitive to the radial extent of the Se p character in the relevant acceptor orbitals. Thus, the combination of covalency, orbital-specific electronic structure, and the surrounding protein environment can give rise to a protonation preference that is not predicted by net atomic charge alone. Consistent with this picture, our protonation calculations identify 2B as the preferred site.

## 4 Conclusion

In this work, we perform QM/MM calculations to understand the experimental observation of the difference in the electronic structure of the belt chalcogenide and establish the electronic origin of site-selective reactivity within the FeMoco cofactor of nitrogenase. The results demonstrate that the apparent symmetry of the belt sulfides is broken by the surrounding protein environment, which imposes distinct electronic structures at the individual belt positions.

Analysis of the calculated spectra shows that the differences in the pre-edge intensities of the belt selenide and sulfide sites cannot be explained by covalency alone but also require consideration of the dipole integral. Our calculations show that the distinct Se and S XAS features between the 2B and 3A/5A sites can be dominantly attributed to the 2B π-transition. The greater 2B π-intensity is partly attributed to the shorter Se/S–Fe bond distances observed crystallographically and by EXAFS, which increase the covalency of their π-bonding. We find that the dominant protein impact stems from noncovalent interactions with hydrophobic residues, localizing the 2B π-lone pair at the center, which increases the dipole integral. Notably, hydrophobic residues like Val70 have been an important mutation site for reactivity studies and have been principally described with steric terms but not as a residue influencing the electronics of the cofactor. Comparatively, H-bonding residues polarize the π-lone pairs away from the cofactor that weakens their covalency.

The localization of electron density at the 2B position directly translates into an increased effective basicity relative to the other belt sulfides. While the dependence of the dipole integral on effective nuclear charge is well established,^31,94,104^ the present results show that the dipole integral is sensitive not only to the charge at a given site, but also to the spatial distribution of electronic charge, i.e., the local charge density. In particular, the dipole integrals are modulated even in the absence of significant changes in the calculated atomic charges, reflecting changes in the radial extent of the relevant valence orbitals. We therefore propose that the dipole integral can provide information about local basicity that is not captured by atomic charge alone. This is consistent with our previous suggestion that the 2B π-lone pair is protonated in the E_1_ state during catalysis.^12^ From a simplified charge-based perspective, one might instead anticipate preferential protonation at the more ionic 3A or 5A positions. However, these sites are engaged in hydrogen-bonding interactions within the protein environment, which likely impose both steric and electronic constraints that disfavor protonation. At the 2B position, the local protein environment instead modulates the spatial distribution of the sulfur-based electron density, altering the radial extent of the valence orbital without a corresponding change in atomic charge. Thus, the site-selective reactivity cannot be understood from charge alone, but depends on how that charge density is distributed and modulated by the surrounding protein environment. These findings provide an electronic rationale for the preference for protonation at S2B and further support prior mechanistic proposals identifying this position as the initial site of protonation during turnover.^11–13^

More broadly, this work demonstrates that secondary-sphere effects in metalloenzymes extend beyond H-bonding interactions and include significant electronic contributions from hydrophobic residues. Such interactions do not merely define the steric environment of the active site, but actively control electron density distribution and reactivity. These insights highlight the importance of considering both hydrophilic and hydrophobic interactions in understanding and designing catalytic metal centers.^112^

## Supplementary Material



## Data Availability

All relevant data generated and analyzed during this study will be made available in the Edmond Open Research Data Repository https://doi.org/10.17617/3.MFQPAQ.

## Notes

Additional figures and tables on computational details and QM/MM model definitions, geometry-optimized conformers (relative energies and Se–Fe distances), Hirshfeld ground-state analysis, localized orbital analysis, TD-DFT Se K-edge XAS spectra, ETS-NOCV pair plots, natural transition orbitals, and excited-state analysis (PDF)
