# Supplementary material for: Secondary-Sphere Control of Electronic Structure and Ligand Basicity in Mo-Nitrogenase: A QM/MM Analysis Guided by Se K‑Edge HERFD XAS
Source: J Am Chem Soc. 2026 Oct 20;148(44):48321–34. doi: 10.1021/jacs.6c16459 (PMC13761202; doi:10.1021/jacs.6c16459)
Supplement: Supplementary file 1 [file ja6c16459_si_001.pdf]

Supporting Information

Secondary-Sphere Control Electronic Structure and Ligand Basicity in Mo-Nitrogenase: A QM/MM Analysis Guided by Se K-edge HERFD XAS

Gursahib Singh Sethi, Justin P. Joyce*, Christina Roemelt, Kushal Sengupta*, Serena DeBeer*

Department of Inorganic Spectroscopy, Max Planck Institute for Chemical Energy Conversion, Mülheim and der Ruhr, Germany
E-mail: justin.joyce@cec.mpg.de, kushal.sengupta@cec.mpg.de, serena.debeer@cec.mpg.de

Table of Contents

1. Se Incorporation in FeMoco 2
2. Computational Details 2
3. Geometry Optimization 4
4. Hirshfeld Ground State Analysis 5
5. Localized orbitals 7
6. TDDFT Se K-edge XAS 8
7. Excited State Analysis 12
8. ETS- NOCV Pair Plots 16
9. Basicity Trend Among the Belt Sites 17
10. References 17

1. Se Incorporation in FeMoco

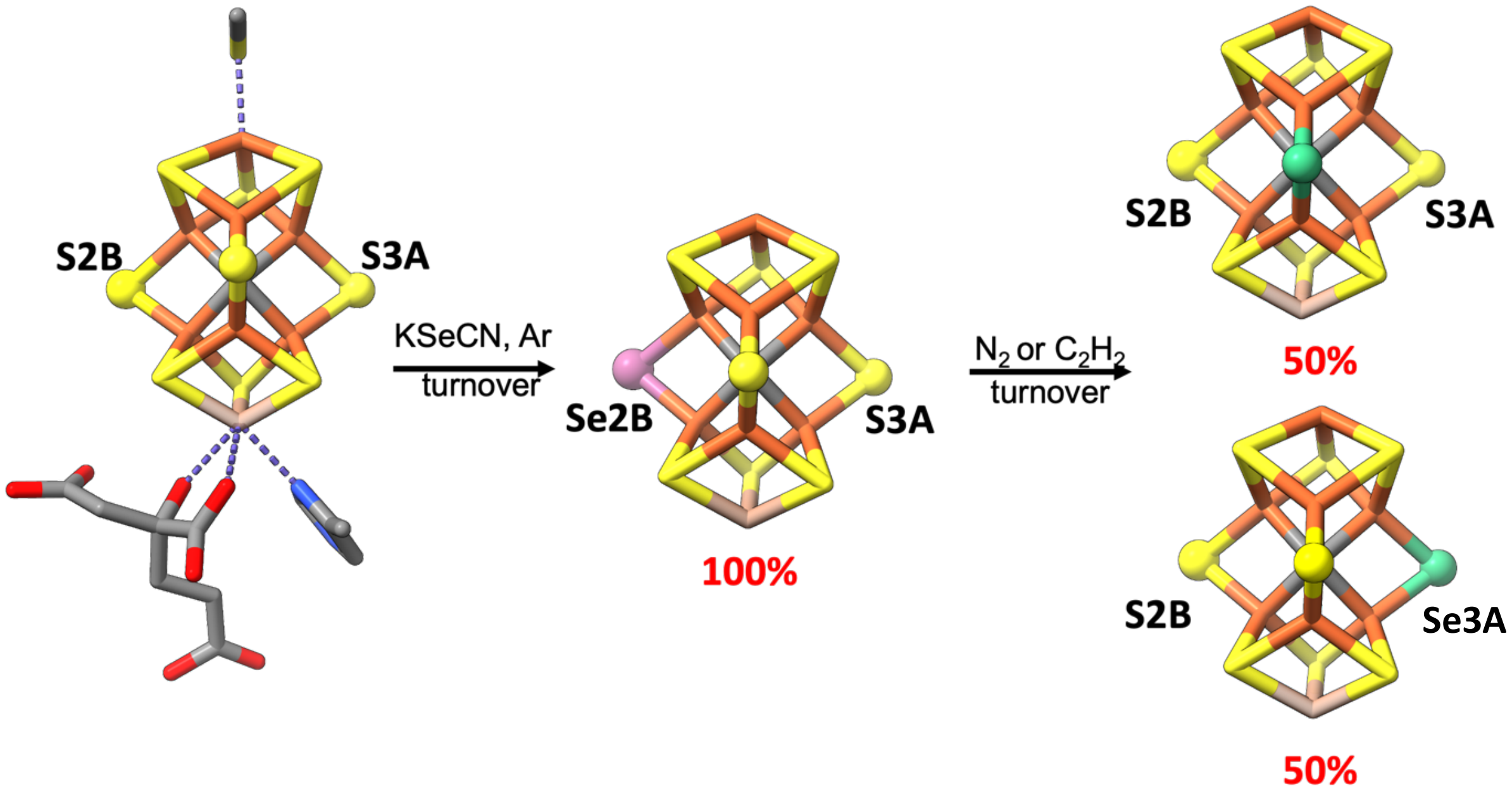

Figure S1. Summary of Se incorporation and migration reactions in FeMoco from *A. vinelandii* (PDB-IDs 3U7Q, 5BVG, 5BVH). The position of Se-incorporated sites is highlighted with different colors: Se2B (pink), Se5A (deep yellow) and Se3A (green). Adapted from *eLife* 2015*, 4,* e11620*.*

2. Computational Details

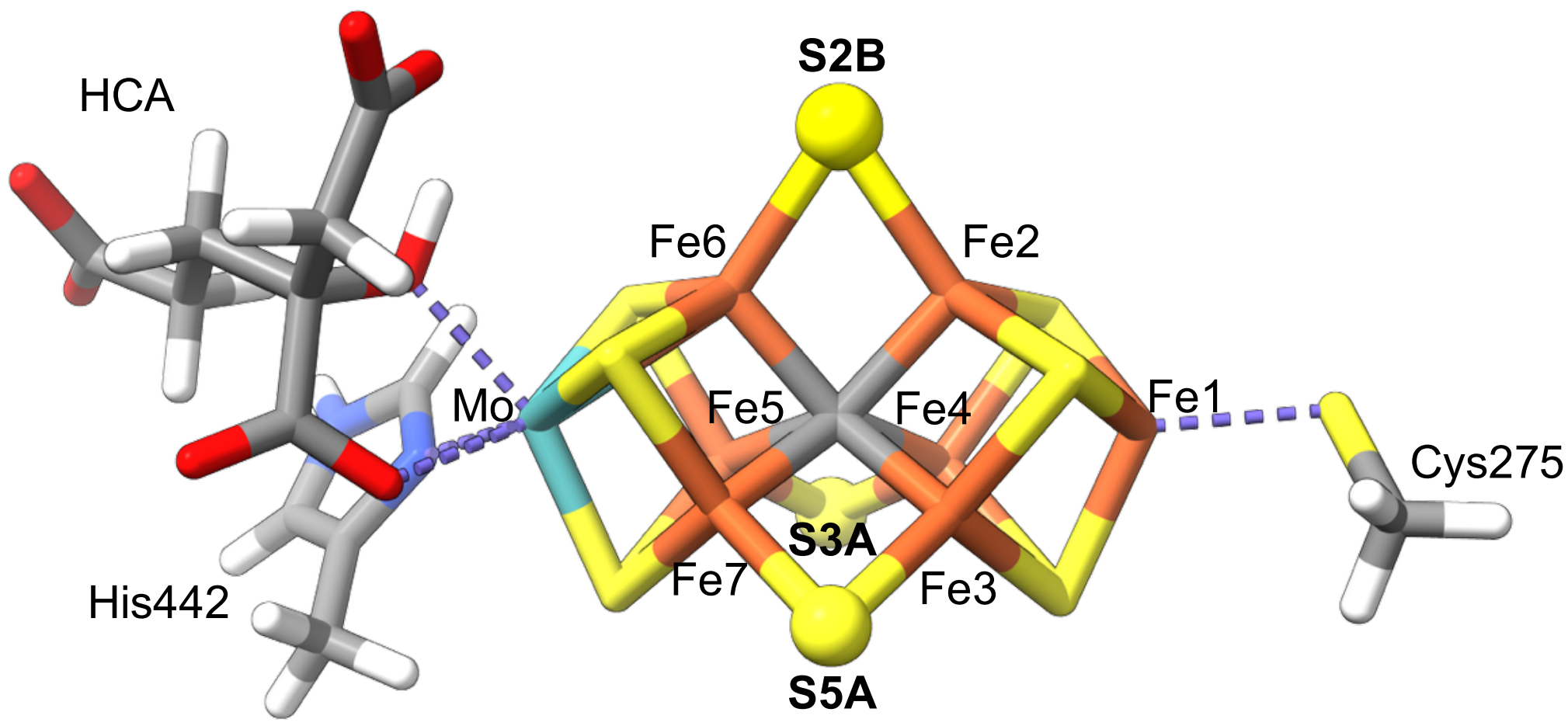

Figure S2. Minimal 56-atom QM region used for both QM and QM(1°)/MM model systems. Contains Cys275, His442, homocitrate (HCA) and wild-type FeMoco.

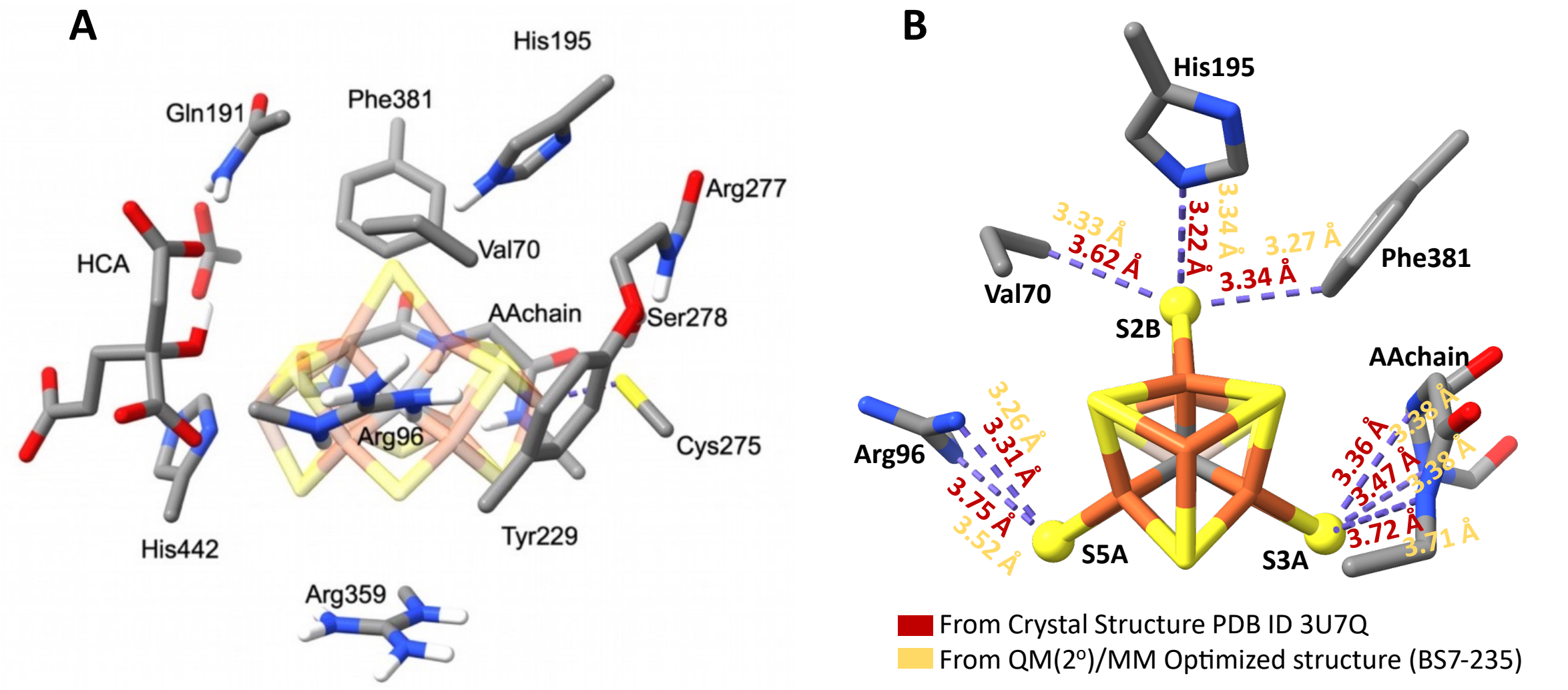

Figure S3. (A) The 191-atom QM region of the QM(2°)/MM model with the labeling scheme indicated. (B) Top-down view of the Fe1–Mo face of the cofactor, showing the H-bonding interaction and associated distance (dark red, crystal structure, PDB ID 3U7Q; yellow, QM(2°)/MM-optimized model, BS7-235 solution). Atom coloring: nitrogen, blue; oxygen, red; carbon, gray. For clarity, the cofactor ligands Cys275, His442, and homocitrate are omitted where necessary.

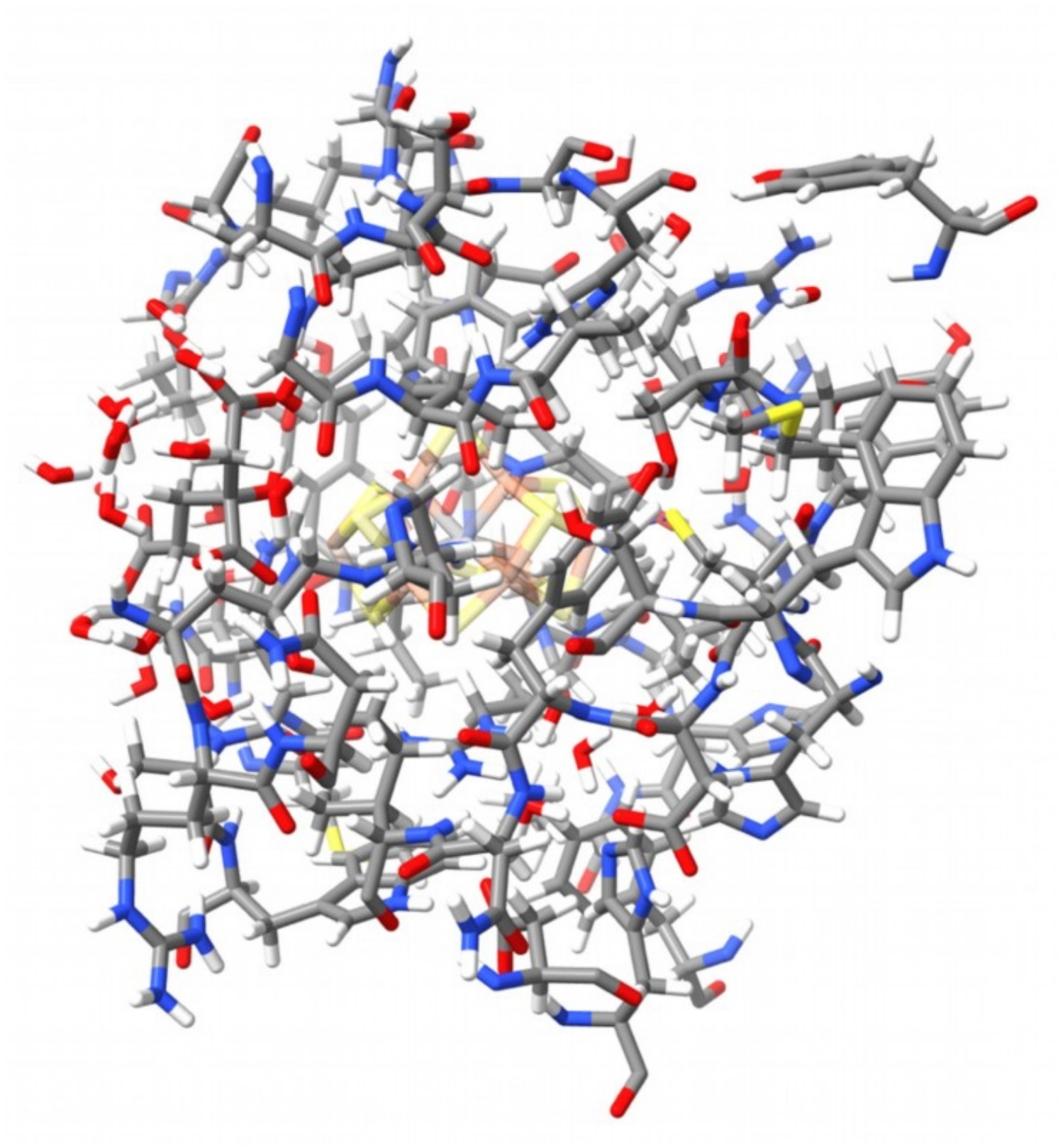

Figure S4. The 990-atom Active region used for both QM(1°)/MM and QM(2°)/MM model.

The active region included 990 atoms, an approximately 10 Å radius with respect to the interstitial carbide. Apart from the 191 atoms from the QM(2°)/MM boundary, the active region contains the following amino acid residues: Thr58, Ile59, Arg60, Gly61, Tyr64, Ala65, Gly66, Ser67, Lys68, Gly69, Val71, Gly73, Pro74, Gly95, Arg97, Asn98, Val189, Ser190, Ser192, Gly194, Ile226, Gly227, Asp228, Asn230, Ile231, Gly233, Asp234, Gln252, Trp253, Ser254, Val273, His274, Tyr276, Met279, Asn280, Tyr281, Phe299, Phe300, Leu353, Tyr354, Pro360, Arg361, His362, Val363, Thr377, Gly378, Tyr379, Ala382, Asp385, Asp386, Gly422, Ser423, Gly424, Glu440, Met441, Ser443, Trp444, Tyr450, and His451. In addition, there are 28 unique water molecules in the active region.

3. Geometry Optimization

BS 7 category with three different spin isomers and BS10 category with ferromagnetic interaction between the Fe of interest were optimized and their respective energies are shown in TableS1.

Table S1. Compilation of the calculated relative energies for the Se-FeMoco. The zero-energy is same between the three Se incorporated conformers.

ΔE (kcal/mol)										
BS	WT	Se2B			Se5A			Se3A		
Soln	QM(2°)/MM	QM	QM(1°)	QM(2°)	QM	QM(1°)	QM(2°)	QM	QM(1°)	QM(2°)
235	0	0.64	0.13	0.08	0.77	0.02	0.14	0.69	0	0
247	0.96	0	1.5	0.74	0	1.31	0.60	0	1.82	1.26
346	0.06	1.03	0	0	1.06	0	0	0.90	0.2	0.19
135	6.99	6.73	7.89	7.35	-	-	-	-	-	-
147	6.45	7.24	6.55	5.92	-	-	-	-	-	-
125	7.07	-	-	-	7.36	6.39	6.71	-	-	-
146	7.06	-	-	-	7.62	6.08	6.68	-	-	-
127	6.51	-	-	-	-	-	-	7.23	6.17	6.29
136	7.78	-	-	-	-	-	-	7.02	7.35	7.59

Table S2. Se−Fe distance (Å) with Se at 2B position

BS Soln	QM			QM(1°)/MM			QM(2°)/MM		
	Se-Fe2	Se-Fe6	$Se\text{-}Fe_{avg}$	Se-Fe2	Se-Fe6	$Se\text{-}Fe_{avg}$	Se-Fe2	Se-Fe6	$Se\text{-}Fe_{avg}$
346	2.37	2.34	2.35	2.35	2.27	2.31	2.33	2.27	2.30
247	2.35	2.36	2.35	2.33	2.28	2.30	2.32	2.29	2.30
235	2.35	2.36	2.35	2.32	2.29	2.30	2.31	2.29	2.30
147	2.38	2.39	2.38	2.36	2.32	2.34	2.34	2.33	2.33
135	2.39	2.39	2.39	2.36	2.31	2.33	2.35	2.32	2.33

Table S3. Se−Fe distance (Å) with Se at 5A position

BS Soln	QM			QM(1°)/MM			QM(2°)/MM		
	Se-Fe3	Se-Fe7	$Se\text{-}Fe_{avg}$	Se-Fe3	Se-Fe7	$Se\text{-}Fe_{avg}$	Se-Fe3	Se-Fe7	$Se\text{-}Fe_{avg}$
346	2.36	2.37	2.36	2.36	2.34	2.35	2.33	2.33	2.33
247	2.37	2.34	2.35	2.38	2.33	2.35	2.36	2.31	2.33
235	2.35	2.37	2.36	2.35	2.34	2.34	2.34	2.33	2.33
146	2.38	2.40	2.39	2.38	2.37	2.37	2.36	2.36	2.36
125	2.38	2.40	2.39	2.38	2.37	2.37	2.36	2.37	2.36

Table S4. Se−Fe distance (Å) with Se at 3A position

BS Soln	QM			QM(1°)/MM			QM(2°)/MM		
	Se-Fe4	Se-Fe5	$Se\text{-}Fe_{avg}$	Se-Fe4	Se-Fe5	$Se\text{-}Fe_{avg}$	Se-Fe4	Se-Fe5	$Se\text{-}Fe_{avg}$
346	2.35	2.37	2.36	2.37	2.43	2.40	2.37	2.36	2.36
247	2.35	2.37	2.36	2.36	2.43	2.39	2.34	2.38	2.36
235	2.37	2.34	2.35	2.39	2.40	2.39	2.35	2.38	2.36
136	2.38	2.40	2.39	2.38	2.46	2.42	2.37	2.42	2.39
127	2.38	2.40	2.39	2.39	2.46	2.42	2.37	2.42	2.39

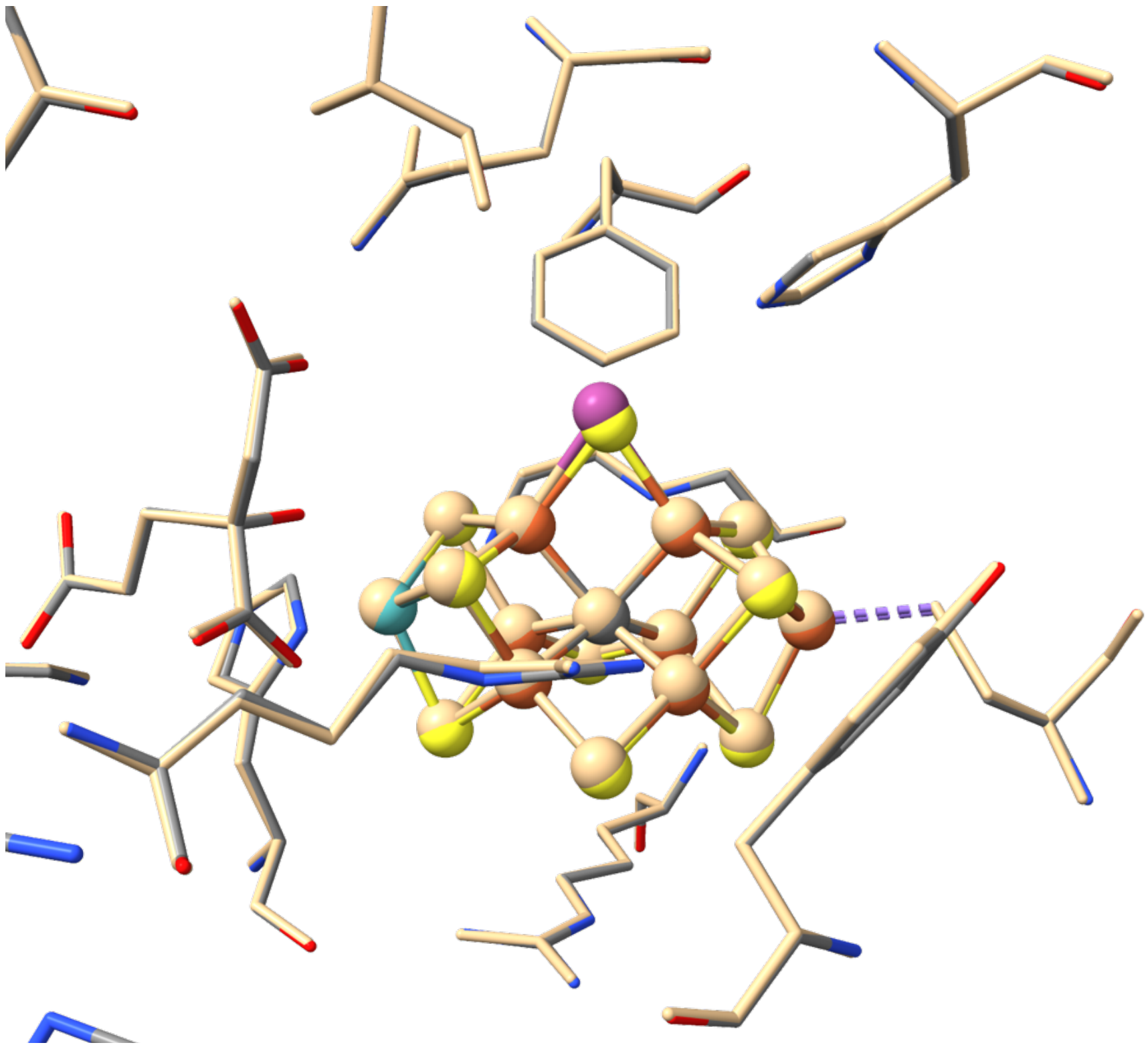

Figure S5. The catalytic active sites of native MoFe (multicolor, PDB: 3U7Q) overlaid with Se-inserted MoFe (single color, PDB: 5BVG). The key active-site residues and cofactor-coordinating side chains superimpose with an RMSD of 0.093 Å, and the hydrogen-bonding partners identified in our QM(2°)/MM model retain the same relative positioning and connectivity in both structures.

4. Hirshfeld Ground State Analysis

Table S5: Calculated Hirshfeld spin populations at the metal centers of the resting-state FeMo-cofactor (WT) and the Se-substituted cofactor, obtained from QM, QM(1°)/MM, and QM(2°)/MM calculations for the BS7-346 broken-symmetry solution. The Hirshfeld charge of the incorporated Se atom is given in the last column.

QM	Mo	Fe1	Fe2	Fe3	Fe4	Fe5	Fe6	Fe7	Se
Se2B	-0.37	3.32	3.17	-3.06	-3.05	2.81	-2.95	2.82	-0.46
Se5A	-0.34	3.33	3.17	-3.08	-3.06	2.81	-2.94	2.83	-0.50
Se3A	-0.34	3.33	3.17	-3.06	-3.08	2.83	-2.94	2.82	-0.50
**QM(1°)/MM**	**Mo**	**Fe1**	**Fe2**	**Fe3**	**Fe4**	**Fe5**	**Fe6**	**Fe7**	**Se**
Se2B	-0.25	3.21	3.11	-2.96	-2.99	2.78	-2.81	2.70	-0.42
Se5A	-0.20	3.20	3.09	-2.97	-2.98	2.78	-2.83	2.70	-0.48
Se3A	-0.20	3.20	3.09	-2.96	-3.00	2.78	-2.83	2.70	-0.51
**QM(2°)/MM**	**Mo**	**Fe1**	**Fe2**	**Fe3**	**Fe4**	**Fe5**	**Fe6**	**Fe7**	**Se**
S	-0.18	3.18	3.08	-2.93	-2.95	2.73	-2.82	2.66	-
Se2B	-0.21	3.18	3.09	-2.93	-2.95	2.73	-2.80	2.67	-0.21
Se5A	-0.17	3.17	3.08	-2.94	-2.95	2.73	-2.83	2.67	-0.38
Se3A	-0.17	3.17	3.08	-2.93	-2.96	2.74	-2.83	2.67	-0.33

Table S6: Calculated Hirshfeld spin populations at the metal centers of the resting-state FeMo-cofactor (WT) and the Se-substituted cofactor, obtained from QM, QM(1°)/MM, and QM(2°)/MM calculations for the BS7-247 broken-symmetry solution. The Hirshfeld charge of the incorporated Se atom is given in the last column.

QM	Mo	Fe1	Fe2	Fe3	Fe4	Fe5	Fe6	Fe7	Se
Se2B	-0.36	3.31	-3.08	3.17	-3.06	2.81	2.84	-2.93	-0.48
Se5A	-0.39	3.32	-3.07	3.18	-3.06	2.82	2.83	-2.94	-0.48
Se3A	-0.36	3.31	-3.06	3.17	-3.07	2.83	2.82	-2.93	-0.50
QM(1°)/MM	Mo	Fe1	Fe2	Fe3	Fe4	Fe5	Fe6	Fe7	Se
Se2B	-0.28	3.21	-2.93	3.12	-2.96	2.80	2.63	-2.87	-0.43
Se5A	-0.32	3.21	-2.91	3.12	-2.96	2.80	2.64	-2.86	-0.47
Se3A	-0.29	3.20	-2.90	3.11	-2.97	2.80	2.64	-2.87	-0.53
QM(2°)/MM	Mo	Fe1	Fe2	Fe3	Fe4	Fe5	Fe6	Fe7	Se
S	-0.30	3.19	-2.89	3.08	-2.90	2.73	2.65	-2.83	-
Se2B	-0.27	3.19	-2.90	3.09	-2.90	2.73	2.63	-2.84	-0.23
Se5A	-0.32	3.19	-2.89	3.09	-2.90	2.73	2.66	-2.83	-0.37
Se3A	-0.28	3.18	-2.89	3.09	-2.92	2.74	2.66	-2.84	-0.33

Table S7: Calculated Hirshfeld spin populations at the metal centers of the resting-state FeMo-cofactor (WT) and the Se-substituted cofactor, obtained from QM, QM(1°)/MM, and QM(2°)/MM calculations for the BS7-235 broken-symmetry solution. The Hirshfeld charge of the incorporated Se atom is given in the last column.

QM	Mo	Fe1	Fe2	Fe3	Fe4	Fe5	Fe6	Fe7	Se
Se2B	-0.48	3.33	-3.08	-3.06	3.17	-2.90	2.85	2.83	-0.48
Se5A	-0.48	3.33	-3.06	-3.08	3.17	-2.90	2.83	2.85	-0.49
Se3A	-0.51	3.32	-3.06	-3.06	3.17	-2.91	2.83	2.84	-0.48
QM(1°)/MM	Mo	Fe1	Fe2	Fe3	Fe4	Fe5	Fe6	Fe7	Se
Se2B	-0.38	3.22	-2.94	-2.95	3.14	-2.89	2.66	2.75	-0.43
Se5A	-0.39	3.22	-2.91	-2.97	3.14	-2.89	2.67	2.75	-0.48
Se3A	-0.42	3.22	-2.91	-2.96	3.15	-2.90	2.67	2.75	-0.50
QM(2°)/MM	Mo	Fe1	Fe2	Fe3	Fe4	Fe5	Fe6	Fe7	Se
S	-0.41	3.20	-2.90	-2.91	3.10	-2.84	2.69	2.69	-
Se2B	-0.39	3.20	-2.92	-2.91	3.10	-2.85	2.67	2.70	-0.22
Se5A	-0.40	3.19	-2.90	-2.92	3.10	-2.85	2.69	2.70	-0.38
Se3A	-0.46	3.20	-2.90	-2.91	3.11	-2.85	2.69	2.71	-0.32

5. Localized orbitals

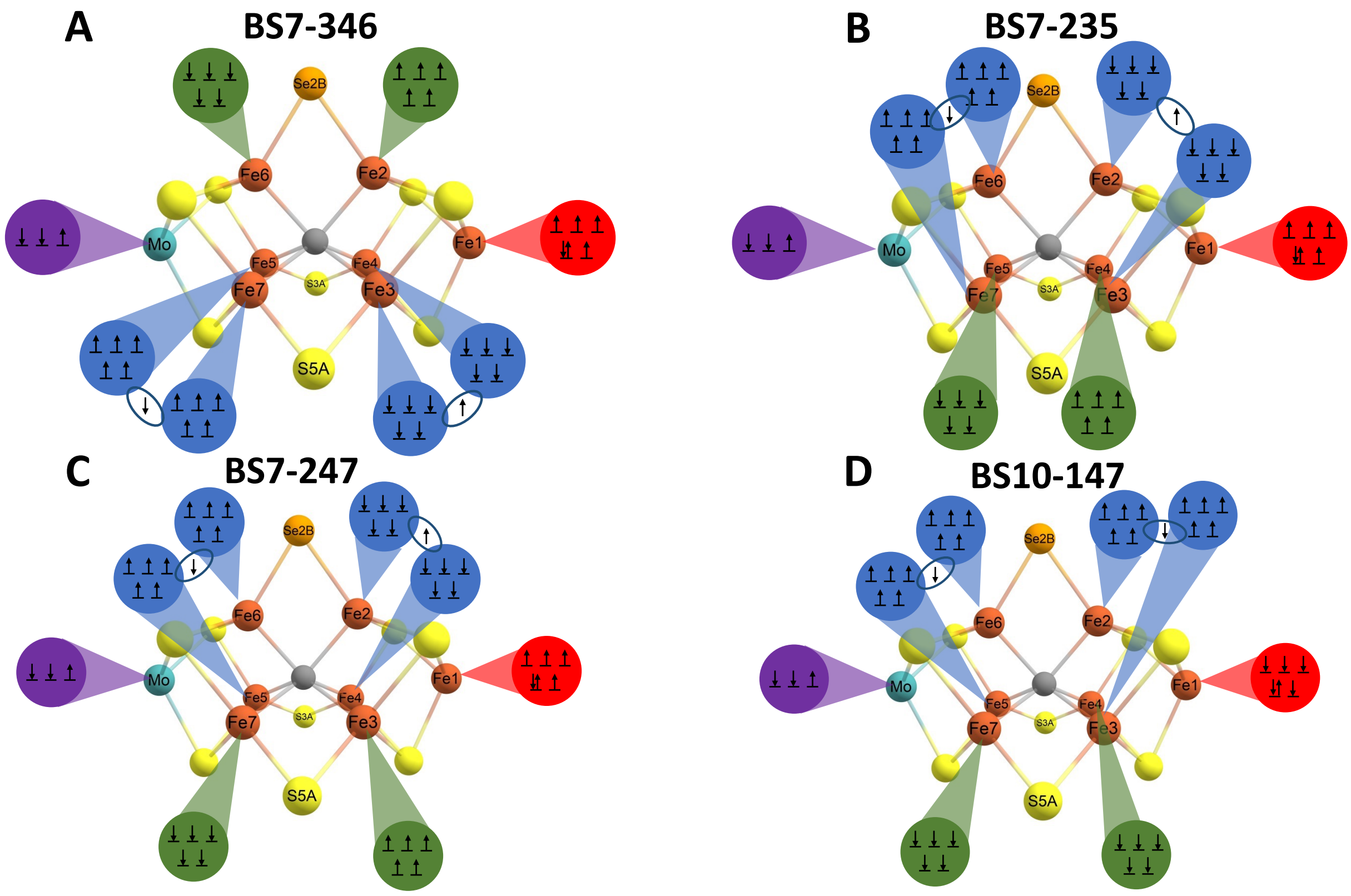

Figure S6. Understanding of the electronic structure of Se-FeMoco (with Se at the 2B site in the QM(2°)/MM model) is based on localized orbital analysis of BS solutions (A) BS7-346, (B) BS7-235, (C) BS7-247, and (D) BS10-147. Delocalized mixed-valent Fe(2.5)–Fe(2.5) pairs are shown in blue. Fe1 remains in the Fe(II) oxidation state (shown in red) in all BS solutions. Fe atoms with a +3-oxidation state are shown in green across all BS solutions.

6. TDDFT Se K-edge XAS

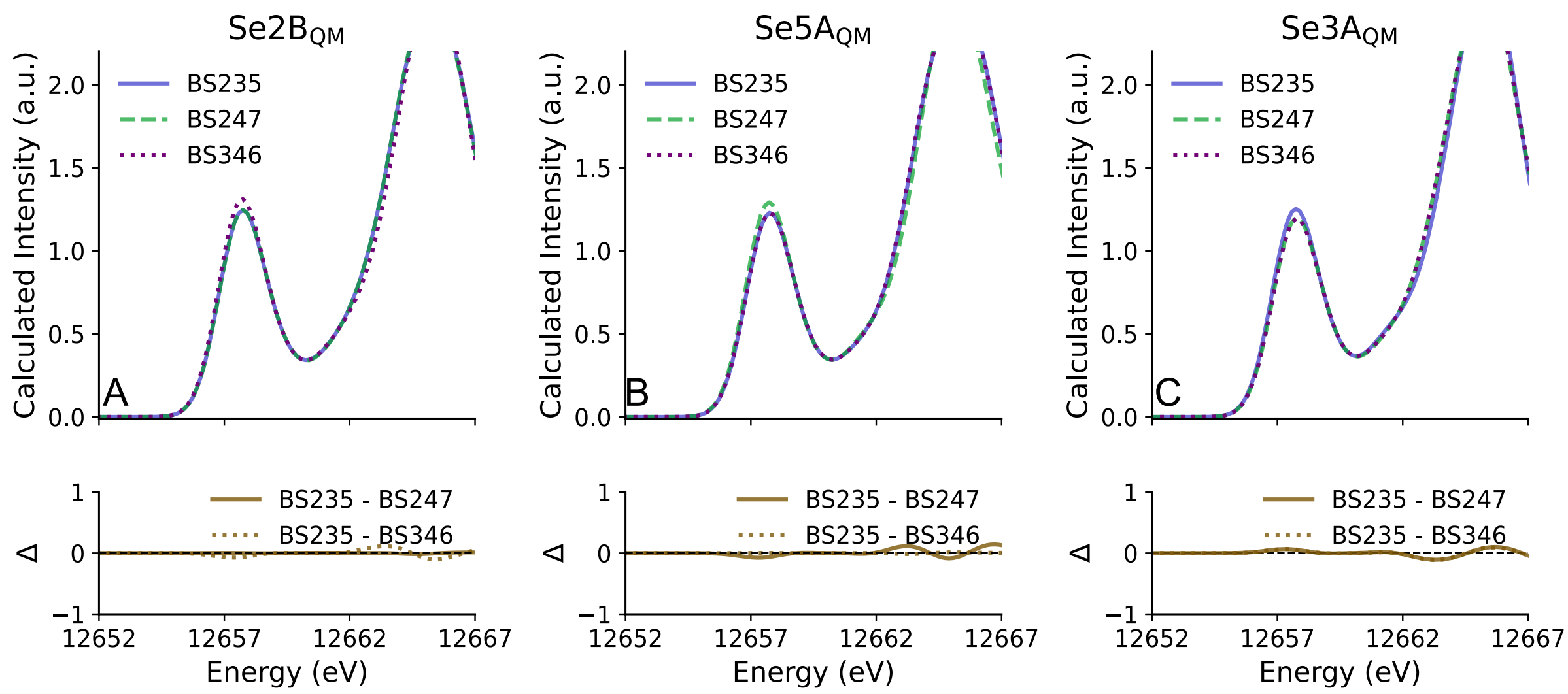

Figure S7. TDDFT-calculated Se K-edge XAS spectra of the three BS7 spin isomers (BS235, blue solid; BS247, green dashed; BS346, red dotted) are shown for the QM model system with Se substitution at (A) Se2B, (B) Se5A, and (C) Se3A. Difference spectra (BS235 − BS247, solid; BS235 − BS346, dotted) are overlaid in brown in each panel to highlight the degree of similarity among the spin isomers. Geometries of the cofactor were independently optimized for each BS solution.

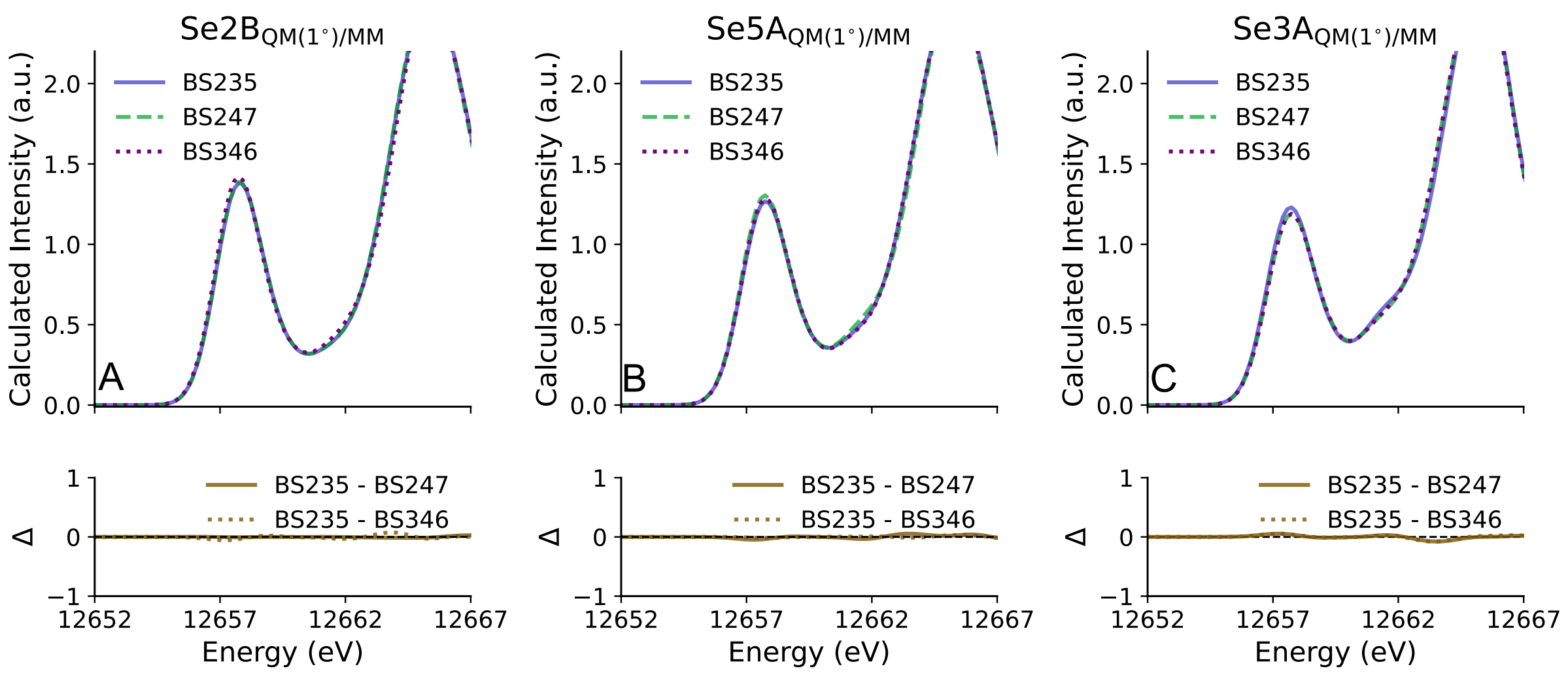

Figure S8. TDDFT-calculated Se K-edge XAS spectra of the three BS7 spin isomers (BS235, blue solid; BS247, green dashed; BS346, red dotted) are shown for the QM(1°)/MM model system with Se substitution at (A) Se2B, (B) Se5A, and (C) Se3A. Difference spectra (BS235 − BS247, solid; BS235 − BS346, dotted) are overlaid in brown in each panel to highlight the degree of similarity among the spin isomers. Geometries of the cofactor were independently optimized for each BS solution.

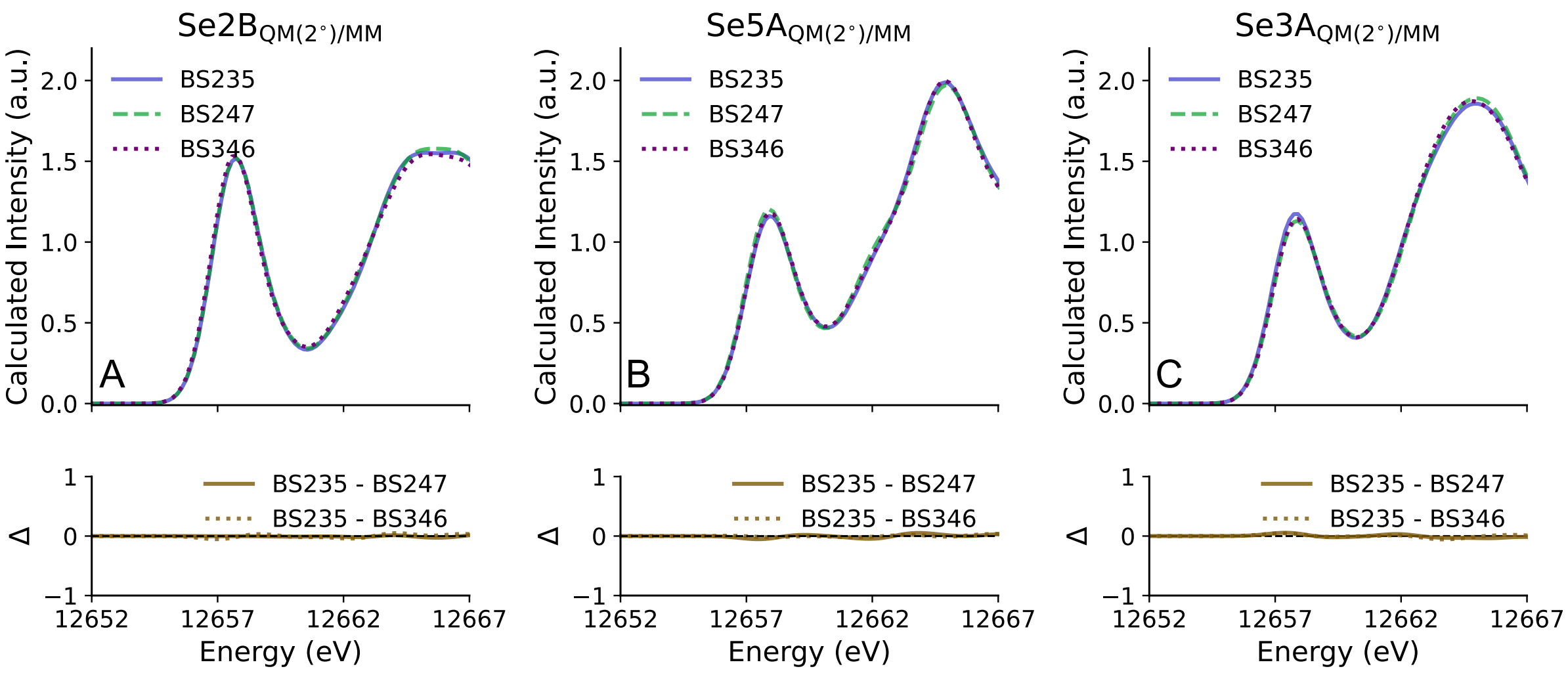

Figure S9. TDDFT-calculated Se K-edge XAS spectra of the three BS7 spin isomers (BS235, blue solid; BS247, green dashed; BS346, red dotted) are shown for the QM(2°)/MM model system with Se substitution at (A) Se2B, (B) Se5A, and (C) Se3A. Difference spectra (BS235 − BS247, solid; BS235 − BS346, dotted) are overlaid in brown in each panel to highlight the degree of similarity among the spin isomers. Geometries of the cofactor were independently optimized for each BS solution.

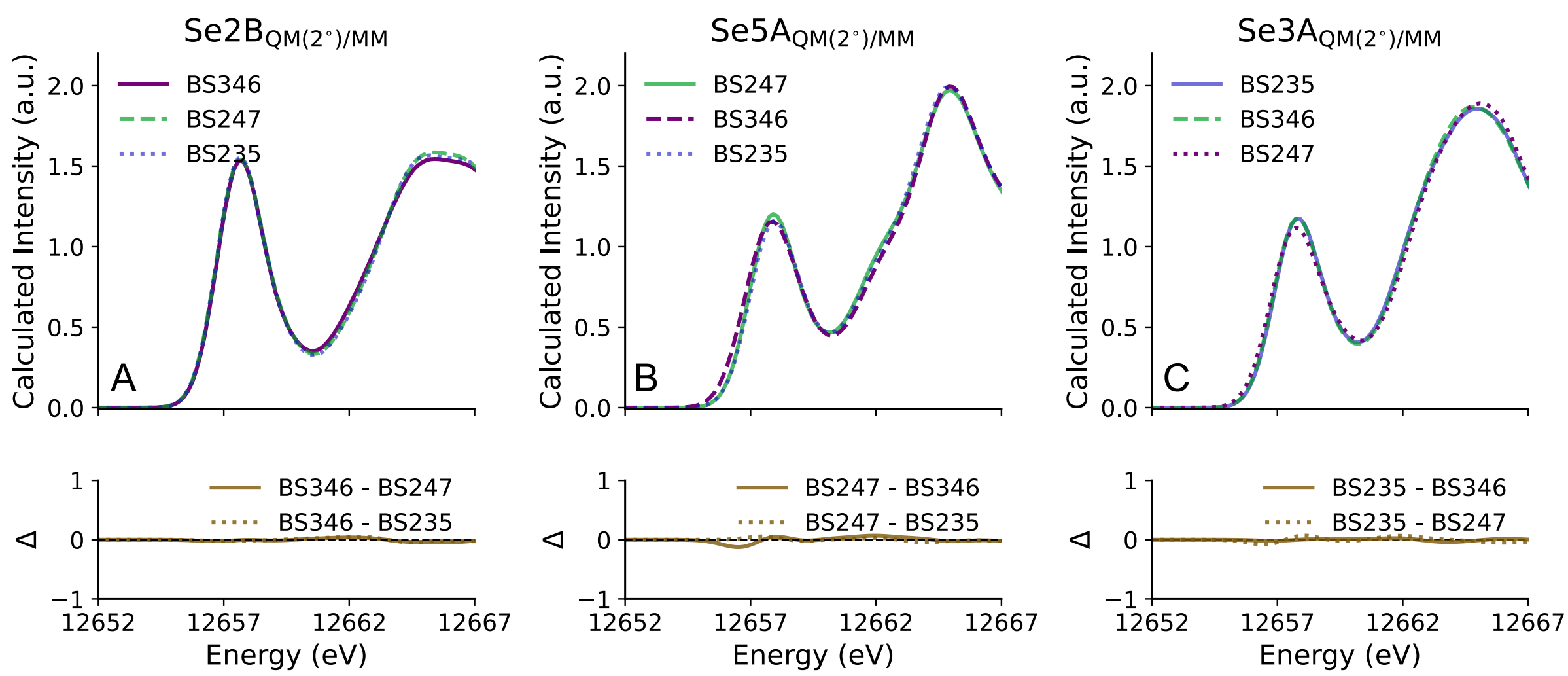

Figure S10. Impact of geometry on keeping the electronic structure constant within different BS solutions for QM(2°)/MM model system. In (A) the electronic structure of BS346 solution is kept constant between the three spin isomers. (B) the electronic structure of BS247 solution in kept constant between the three spin isomers. (C) the electronic structure of BS235 solution in kept constant between the three spin isomers. The difference between the spin isomers is as shown below using brown curve.

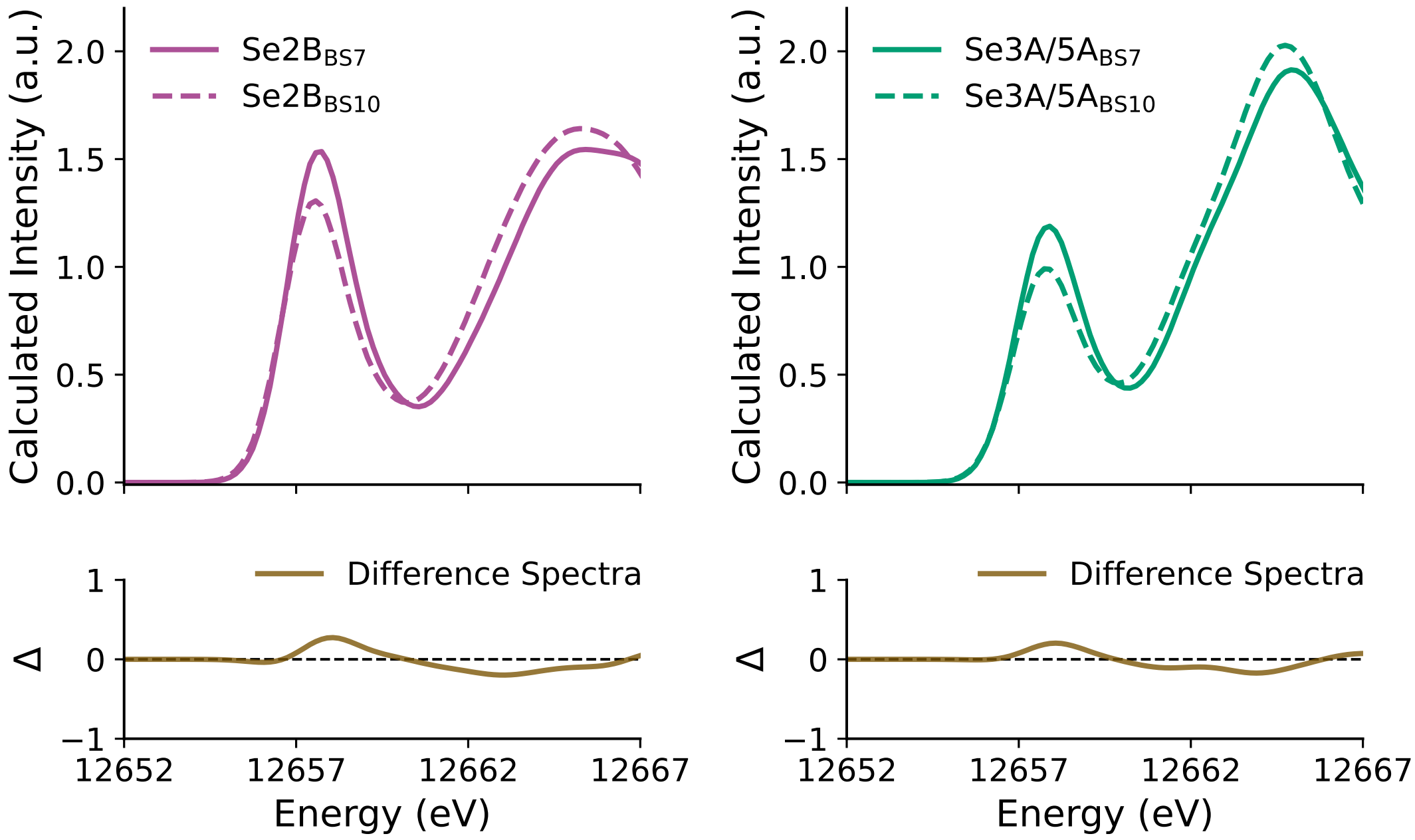

Figure S11. TDDFT-calculated Se K-edge XAS spectra for QM(2°)/MM with Se at (a) 2B (pink) and (B) 3A/5A (green) calculated for the BS7 (solid line) and BS10 (dashed line) solutions. Difference spectra (BS7-BS10) are plotted in brown to illustrate site-specific spectral distinctions.

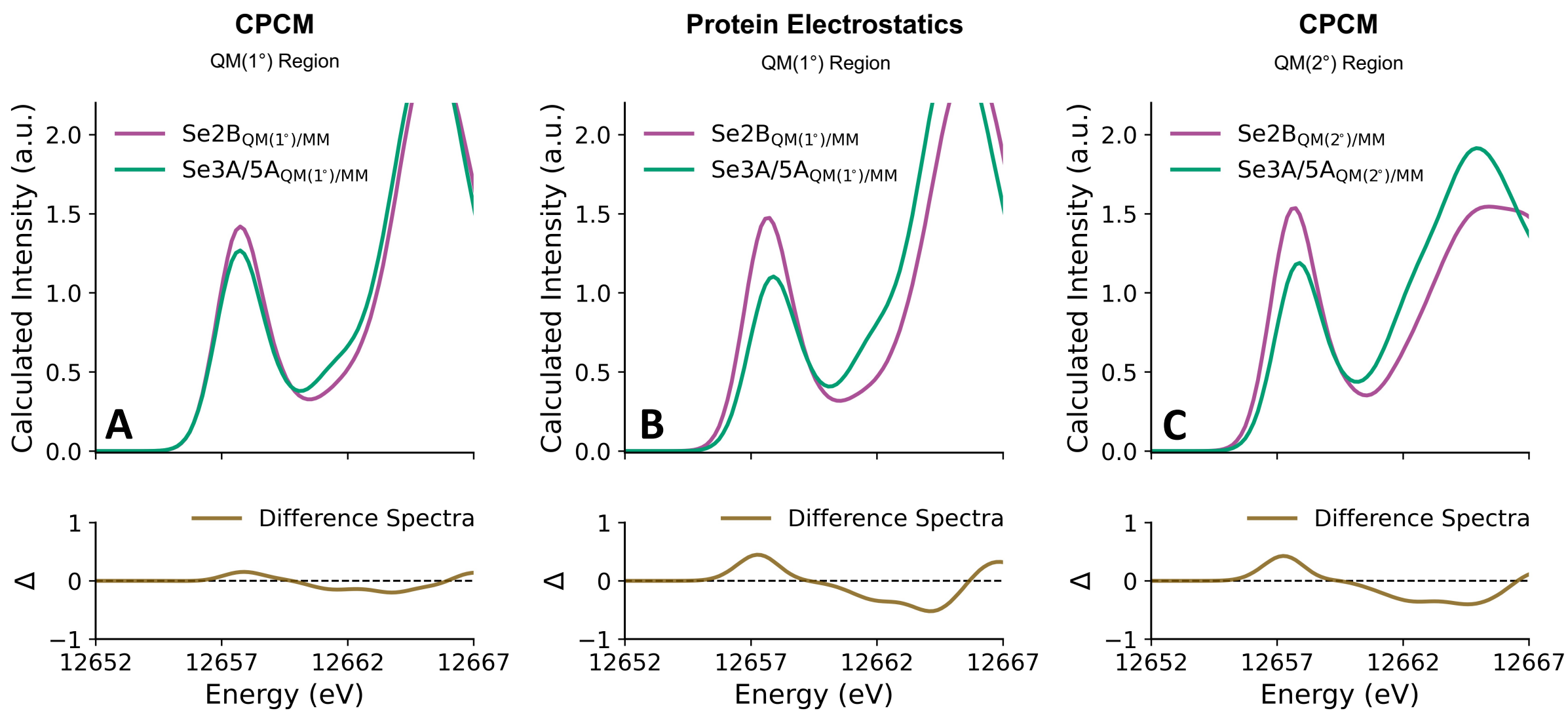

Figure S12. TD-DFT calculated Se K-edge XAS spectra for (A) **QM(1°)/MM** model, (B) **QM(1°)/MM** model including the electrostatic protein environment as point charges, and (C) **QM(2°)/MM**). The difference spectra of Se2B with respect to Se3A/5A are plotted in brown for each panel.

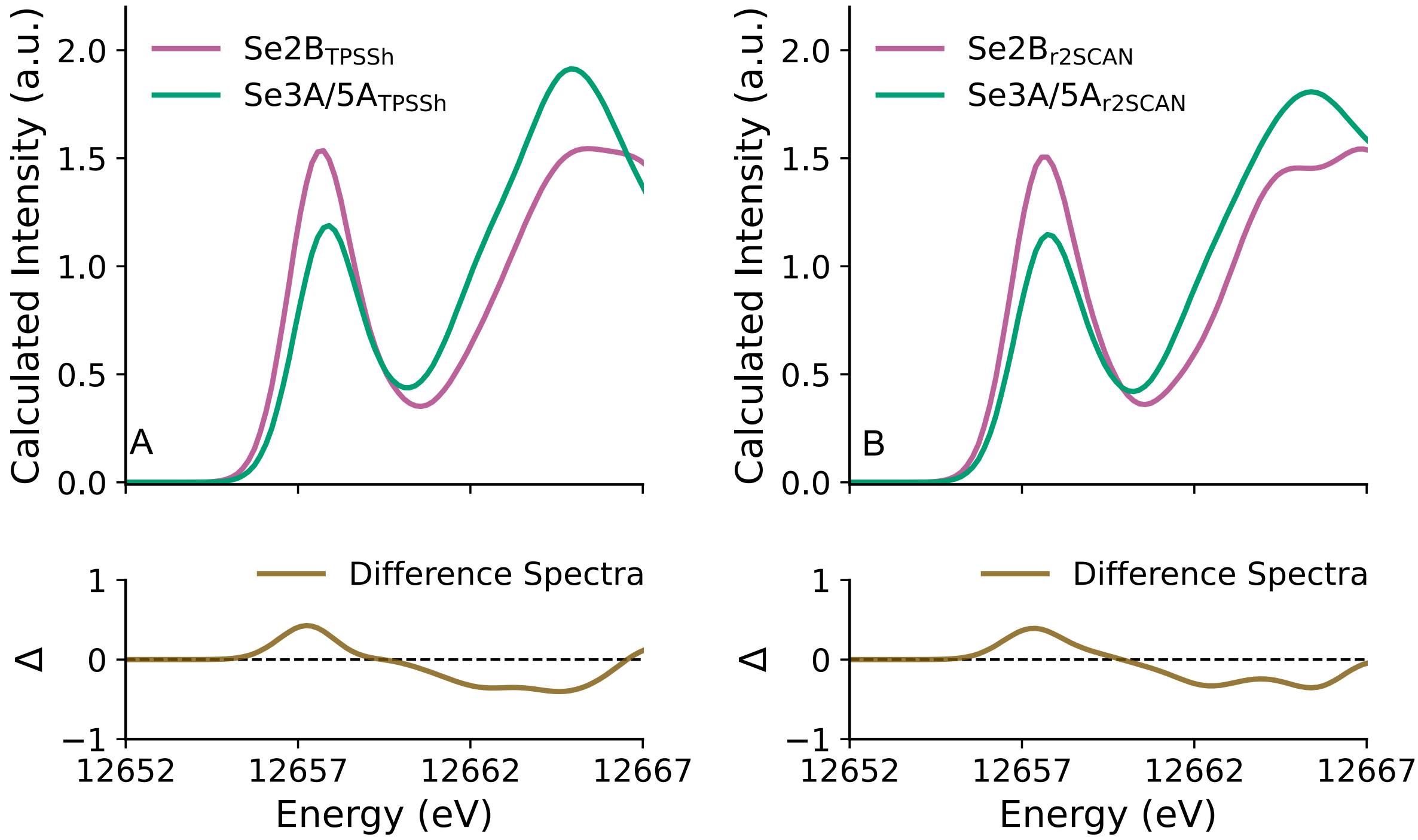

Figure S13. TDDFT-calculated Se K-edge XAS spectra for QM(2°)/MM with Se at 2B (pink) and 3A/5A (green) calculated using (A) TPSSh and (B) r^2SCAN exchange-correlation functionals respectively. Difference spectra (Se2B-Se3A/5A) are plotted in brown to illustrate site-specific spectral distinctions.

Table S8. Pre-edge Energy and area under the curve for all different Se XAS spectra. Calculated values are for TPSSh functional. Unless otherwise stated, calculations were performed on the optimized QM region using CPCM, without electrostatic embedding.

**Model**	**Se site**	**Area**	**Diff Area %**
Exp	Se2B	3.56 ± 0.04	46.91
	Se3A/5A	1.89 ± 0.09	
QM	Se2B	3.22 ± 0.03	2.50
	Se3A/5A	3.14 ± 0.03	
QM(1°)/MM	Se2B	3.44 ± 0.03	9.00
	Se3A/5A	3.13 ± 0.03	
QM(1°)/MM (Protein Electrostatics)	Se2B	3.63 ± 0.04	24.52
	Se3A/5A	2.74 ± 0.03	
QM(2°)/MM	Se2B	3.74 ± 0.03	21.92
	Se3A/5A	2.92 ± 0.02	
**Model**	**S site**	**Area**	**Diff Area %**
WT-QM(2°)/MM	S2B	22.50 ± 0.18	19.60
	S3A/5A	18.09 ± 0.14	

7. Excited State Analysis

Table S9: Calculated transition energies, oscillator strengths, and Se 4p orbital contributions (Hirshfeld population analysis) for the relevant donor–acceptor transitions for QM model.

Transition	$Se2B_{QM}$			$Se5A_{QM}$			$Se3A_{QM}$		
	Energy(eV)	f_{osc} (10^{-5})	% Se	Energy(eV)	f_{osc} (10^{-5})	% Se	Energy(eV)	f_{osc} (10^{-5})	% Se
**σ_α**	12657.30	7.82	8.98	12657.32	7.91	9.46	12657.32	7.66	9.14
**σ_β**	12657.39	8.78	9.69	12657.39	8.64	9.77	12657.38	8.26	9.28
**π_α**	12657.70	7.98	12.02	12657.76	7.45	11.54	12657.79	5.78	9.38
**π_β**	12657.83	6.52	10.01	12657.83	7.02	10.84	12657.82	6.40	9.92

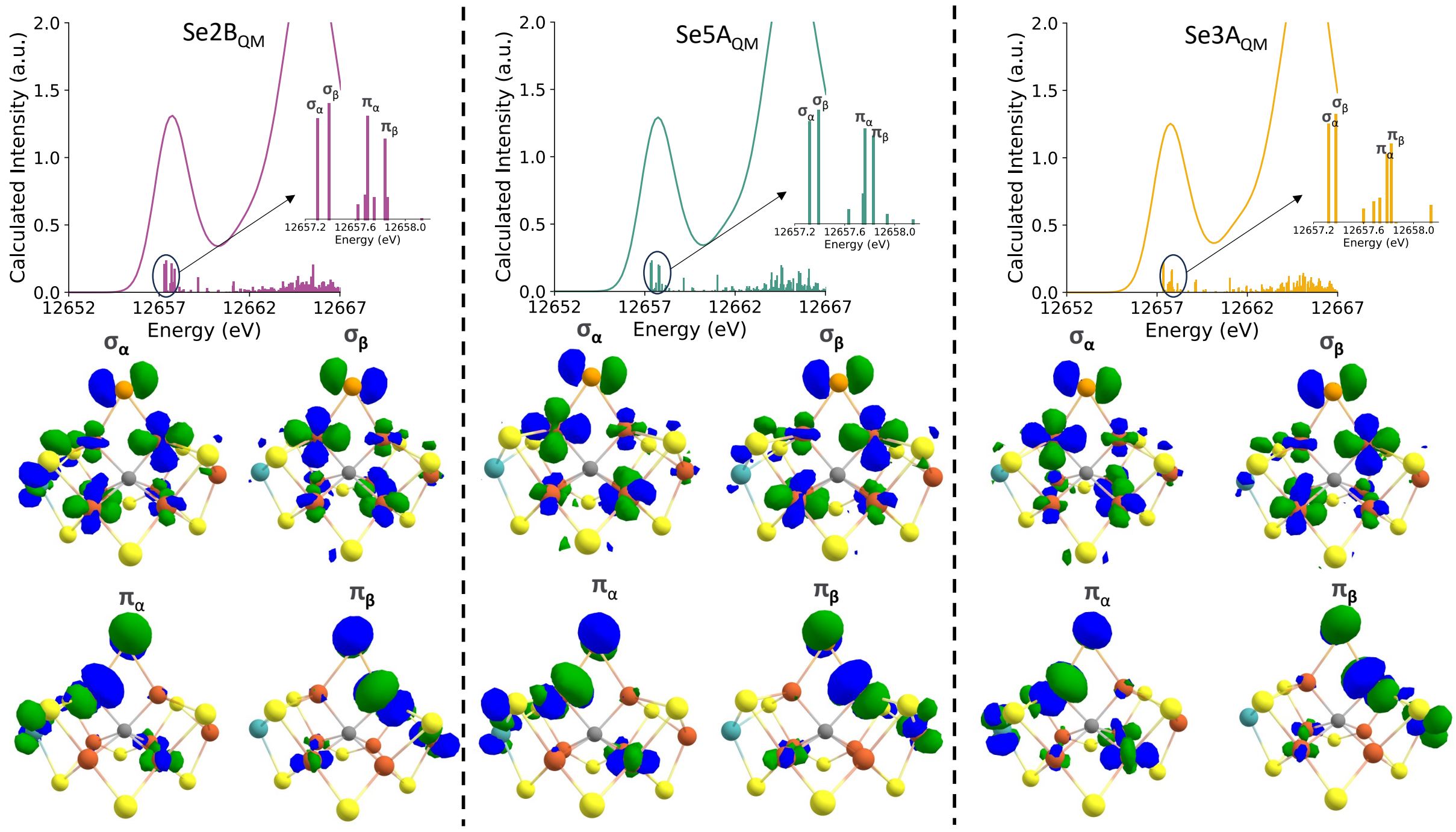

Figure S14. Natural transition orbitals (NTOs) corresponding to the most intense calculated transitions for Se incorporation at the 2B (left), 5A (middle), and 3A (right) sites within the QM cluster model. The α and β notation refers to the spin of the transition.

Table S10: Calculated transition energies, oscillator strengths, and Se 4p orbital contributions (Hirshfeld population analysis) for the relevant donor–acceptor transitions for QM(1°)/MM model.

Transition	$Se2B_{QM(1°)/MM}$			$Se5A_{QM(1°)/MM}$			$Se3A_{QM(1°)/MM}$		
	Energy(eV)	f_{osc} (10^{-5})	% Se	Energy(eV)	f_{osc} (10^{-5})	% Se	Energy(eV)	f_{osc} (10^{-5})	% Se
**σ_α**	12657.38	8.46	9.91	12657.34	7.88	9.18	12657.30	7.14	8.50
**σ_β**	12657.46	9.00	10.21	12657.42	9.10	10.22	12657.35	8.73	9.85
**$\pi_{\alpha1}$**	12657.66	7.40	10.99	12657.69	7.60	11.56	12657.70	6.16	9.43
**$\pi_{\alpha2}$**	12657.70	3.82	6.41	-	-	-	-	-	-
**π_β**	12657.89	7.14	10.85	12657.89	6.39	10.00	12657.81	5.94	9.34

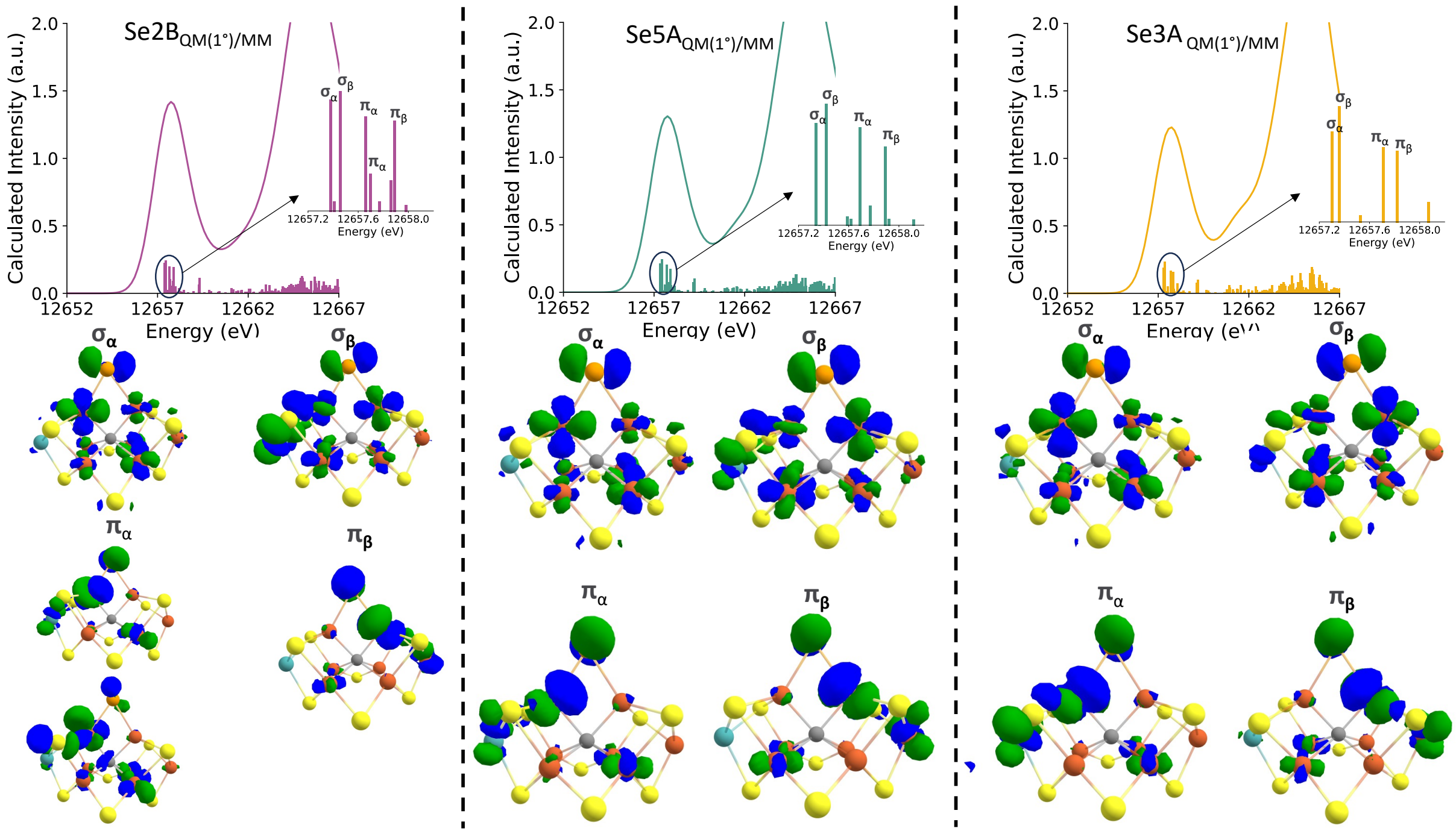

Figure S15. Natural transition orbitals (NTOs) corresponding to the most intense calculated transitions for Se incorporation at the 2B (left), 5A (middle), and 3A (right) sites within the QM(1°)/MM model system. The α and β notation refers to the spin of the transition.

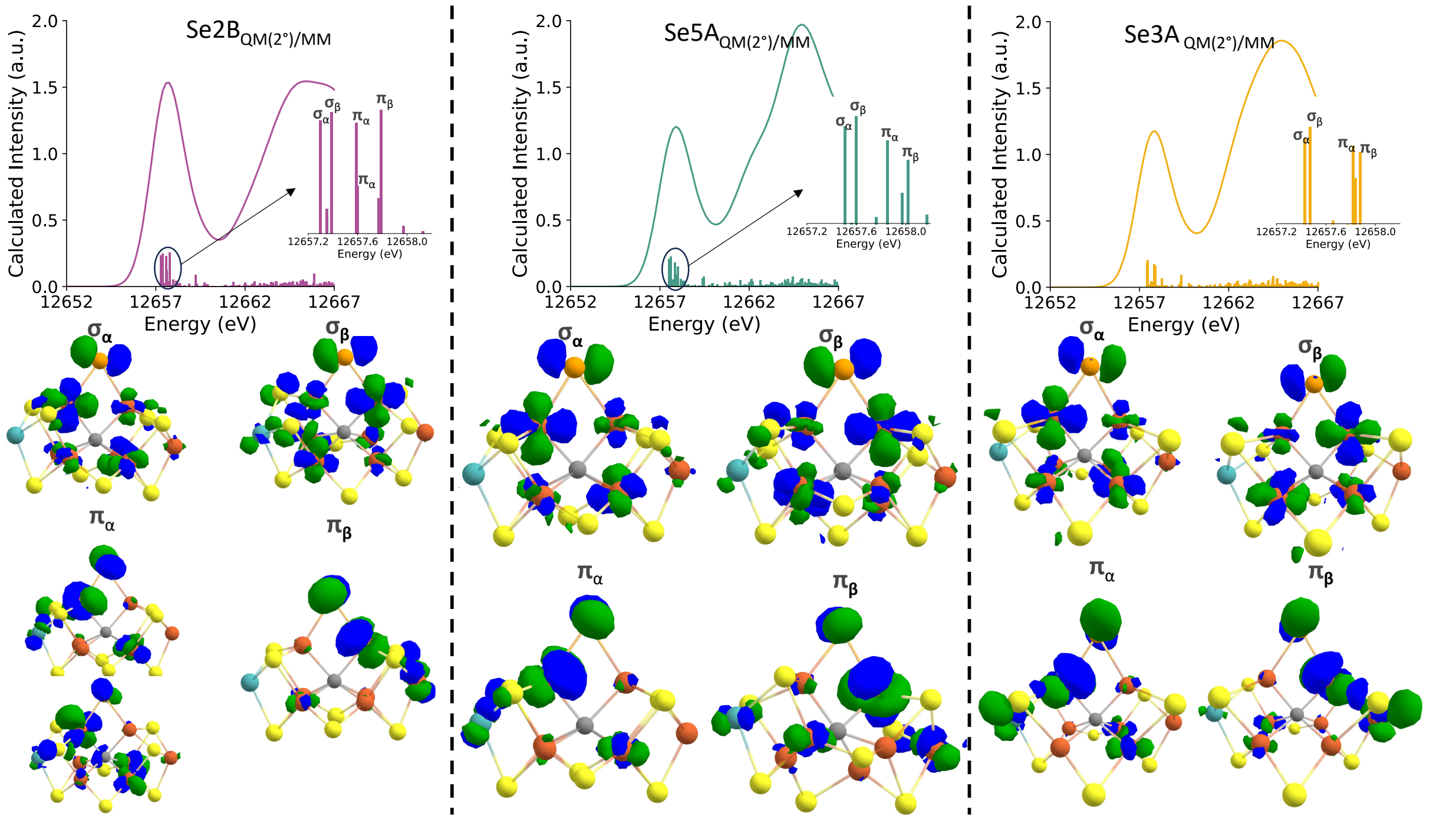

Figure S16. Natural transition orbitals (NTOs) corresponding to the most intense calculated transitions for Se incorporation at the 2B (left), 5A (middle), and 3A (right) sites within the QM(2°)/MM model system. The α and β notation refers to the spin of the transition.

Table S11: Calculated transition energies, oscillator strengths, and Se 4p orbital contributions (Hirshfeld population analysis) for the relevant donor–acceptor transitions for QM(2°)/MM model.

Transition	$Se2B_{QM(2°)/MM}$			$Se5A_{QM(2°)/MM}$			$Se3A_{QM(2°)/MM}$		
	Energy(eV)	f_{osc} (10^{-5})	% Se	Energy(eV)	f_{osc} (10^{-5})	% Se	Energy(eV)	f_{osc} (10^{-5})	% Se
σ_α	12657.30	8.67	9.09	12657.51	7.65	8.59	12657.43	6.88	7.65
σ_β	12657.39	9.19	9.20	12657.60	8.31	8.95	12657.47	7.64	8.11
$\pi_{\alpha1}$	12657.59	8.52	10.40	12657.85	6.75	9.88	12657.82	6.45	9.12
$\pi_{\alpha2}$	12657.60	4.52	6.15	-	-	-	-	-	-
π_β	12657.79	9.59	11.11	12658.02	5.51	8.11	12657.94	6.04	8.34

Table S12: Calculated transition energies, oscillator strengths, and Se 4p orbital contributions (Hirshfeld population analysis) for the relevant donor–acceptor transitions for QM(2°)/MM model on removal of hydrogen bonding residues from the respective Se occupied sites.

Transition	$Se2B_{w/oHis195}$			$Se5A_{w/oArg96}$			$Se3A_{w/oAA}$		
	Energy(eV)	f_{osc} (10^{-5})	% Se	Energy(eV)	f_{osc} (10^{-5})	% Se	Energy(eV)	f_{osc} (10^{-5})	% Se
σ_α	12657.27	8.97	9.63	12657.35	7.99	9.10	12657.30	7.78	8.94
σ_β	12657.37	9.70	10.19	12657.44	8.57	9.17	12657.34	8.65	9.27
$\pi_{\alpha1}$	12657.55	8.07	10.01	12657.73	7.52	10.97	12657.71	6.84	10.01
$\pi_{\alpha2}$	12657.57	6.27	7.88	-	-	-	-	-	-
π_β	12657.73	10.40	11.78	12657.87	6.33	9.49	12657.76	6.78	9.71

Table S13: Calculated transition energies, oscillator strengths, and Se 4p orbital contributions (Hirshfeld population analysis) for the relevant donor–acceptor transitions for Se2B QM(2°)/MM model on removal of respective amino acid residues.

Transition	$Se2B_{w/oVal70}$			$Se2B_{w/oPhe381}$			$Se2B_{w/oAA}$		
	Energy(eV)	f_{osc} (10^{-5})	% Se	Energy(eV)	f_{osc} (10^{-5})	% Se	Energy(eV)	f_{osc} (10^{-5})	% Se
σ_α	12657.31	8.80	9.36	12657.31	8.85	9.33	12657.33	8.53	9.01
σ_β	12657.40	9.40	9.45	12657.40	9.32	9.38	12657.41	8.98	9.03
$\pi_{\alpha1}$	12657.61	5.81	8.04	12657.61	2.55	4.49	12657.61	10.27	12.17
$\pi_{\alpha2}$	12657.62	6.26	8.77	12657.64	9.81	12.39	12657.81	6.52	8.10
π_β	12657.81	8.42	11.05	12657.82	8.97	11.29	12657.79	6.06	7.21
	$Se2B_{w/oArg359}$			$Se2B_{w/oGln191}$			$Se2B_{w/oGlu380}$		
σ_α	12657.33	8.52	9.04	12657.30	8.70	9.25	12657.31	8.64	9.02
σ_β	12657.41	9.01	9.13	12657.39	9.15	9.22	12657.40	9.18	9.21
$\pi_{\alpha1}$	12657.61	9.18	11.13	12657.59	7.79	9.76	12657.57	9.32	11.11
$\pi_{\alpha2}$	12657.62	3.16	4.88	12657.60	5.14	6.79	12657.61	3.46	5.15
π_β	12657.81	8.96	10.61	12657.78	9.16	10.73	12657.82	9.34	10.94

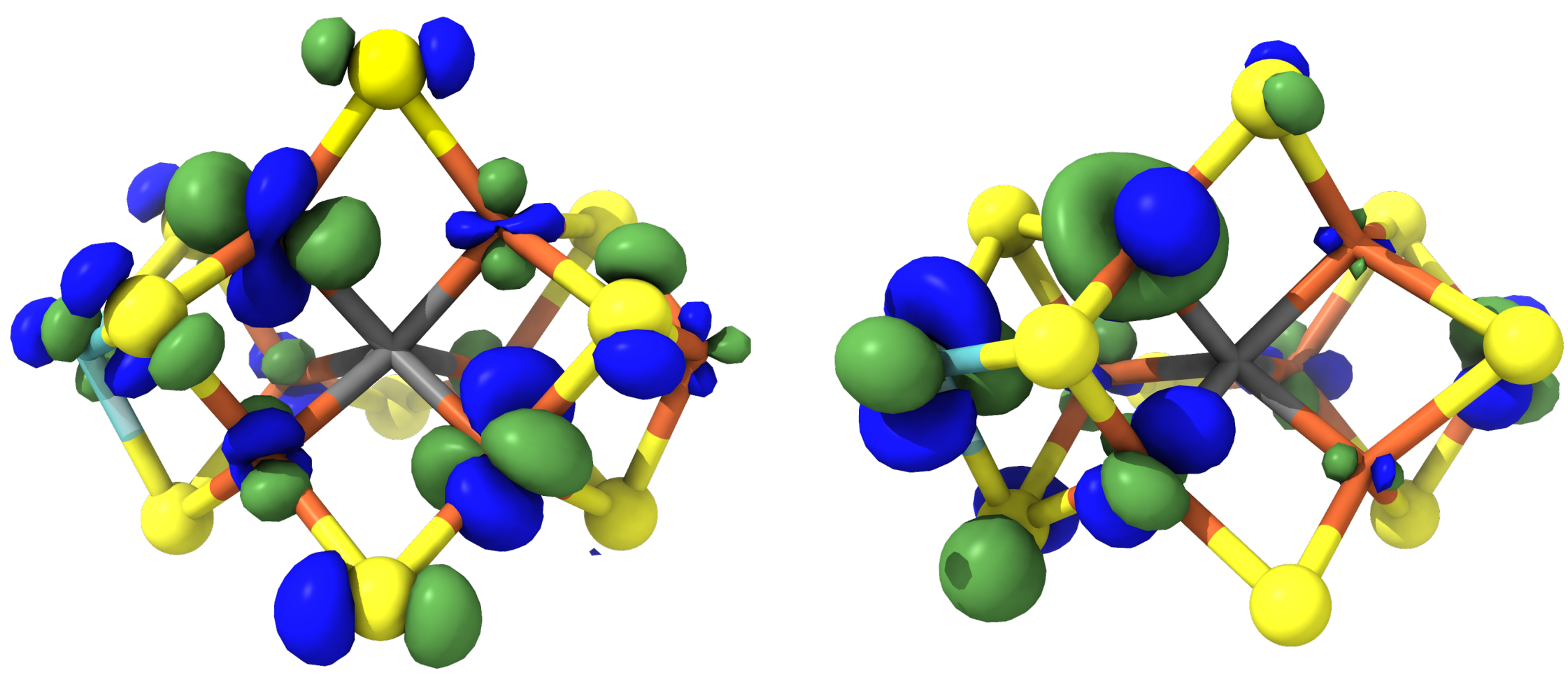

Figure S17. Natural transition orbitals (NTOs) corresponding to the most intense calculated transitions for WT-FeMoco showing that more than one S atom contributes to a given σ- or π-type interaction simultaneously.

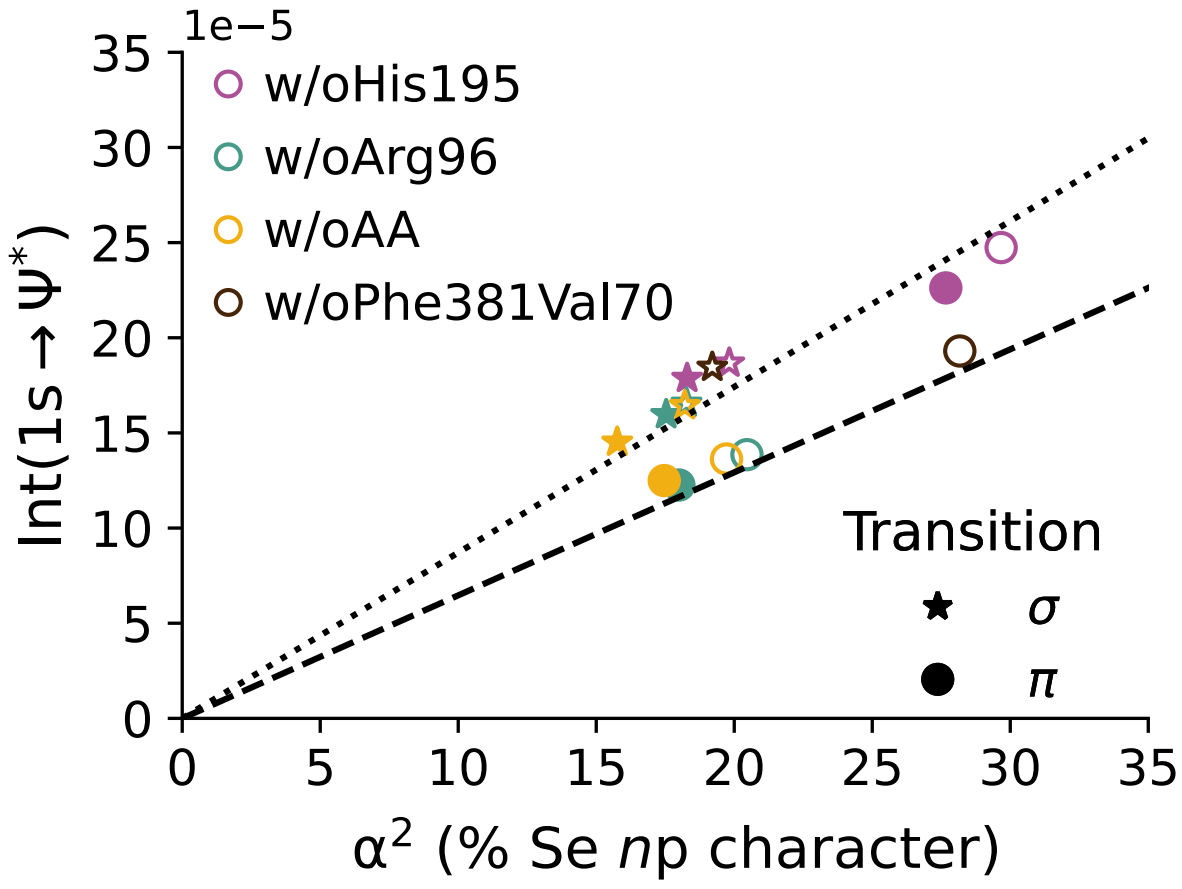

Figure S18. TDDFT-calculated oscillator strengths (Int(1s → ψ*)) are plotted against the %Se np character (α^2 , covalency) per hole for both σ (star markers) and π (circle markers) transitions for QM2°)/MM with (solid) and without (hollow) AA residues of interest with Se at 2B (pink), 5A (green) and 3A (deep yellow). The solid markers correspond to the values taken from Figure 5D of the main text. Trendlines for the pre-edge transitions are carried over from Figure 5 in the main text: dotted for σ and dashed line for π.

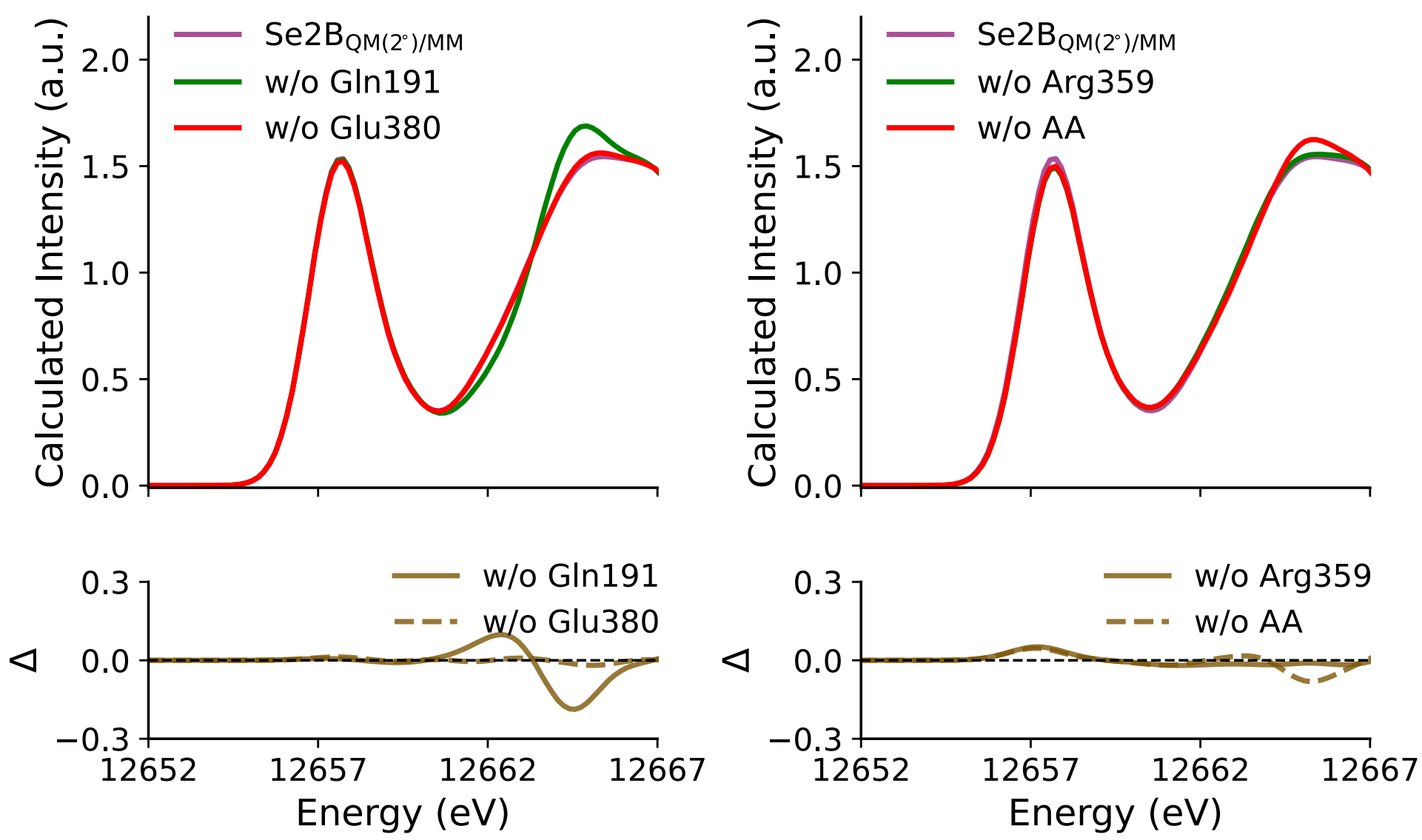

Figure S19. TDDFT-calculated Se XAS spectra for the QM(2°)/MM model system with Se at 2B position. The two plots show the impact of nearby residues on the XAS spectra on removing them from the whole cluster. The plot in pink represents the intact system (with all the secondary sphere residues) compared with removing Gln191 (green) and Glu380 (red) in the left and Arg96 (green) and Gly356/Gly357 (red) in the right. The Difference spectra (intact minus “without residue”), plotted in brown indicate that omission of Gln191 and Glu380 does not impact the pre-edge intensity whereas omission of Arg359 and AA residues including Ile355, Gly356, Gly357, and Leu358 does show a change in the pre-edge intensity.

8. ETS- NOCV Pair Plots

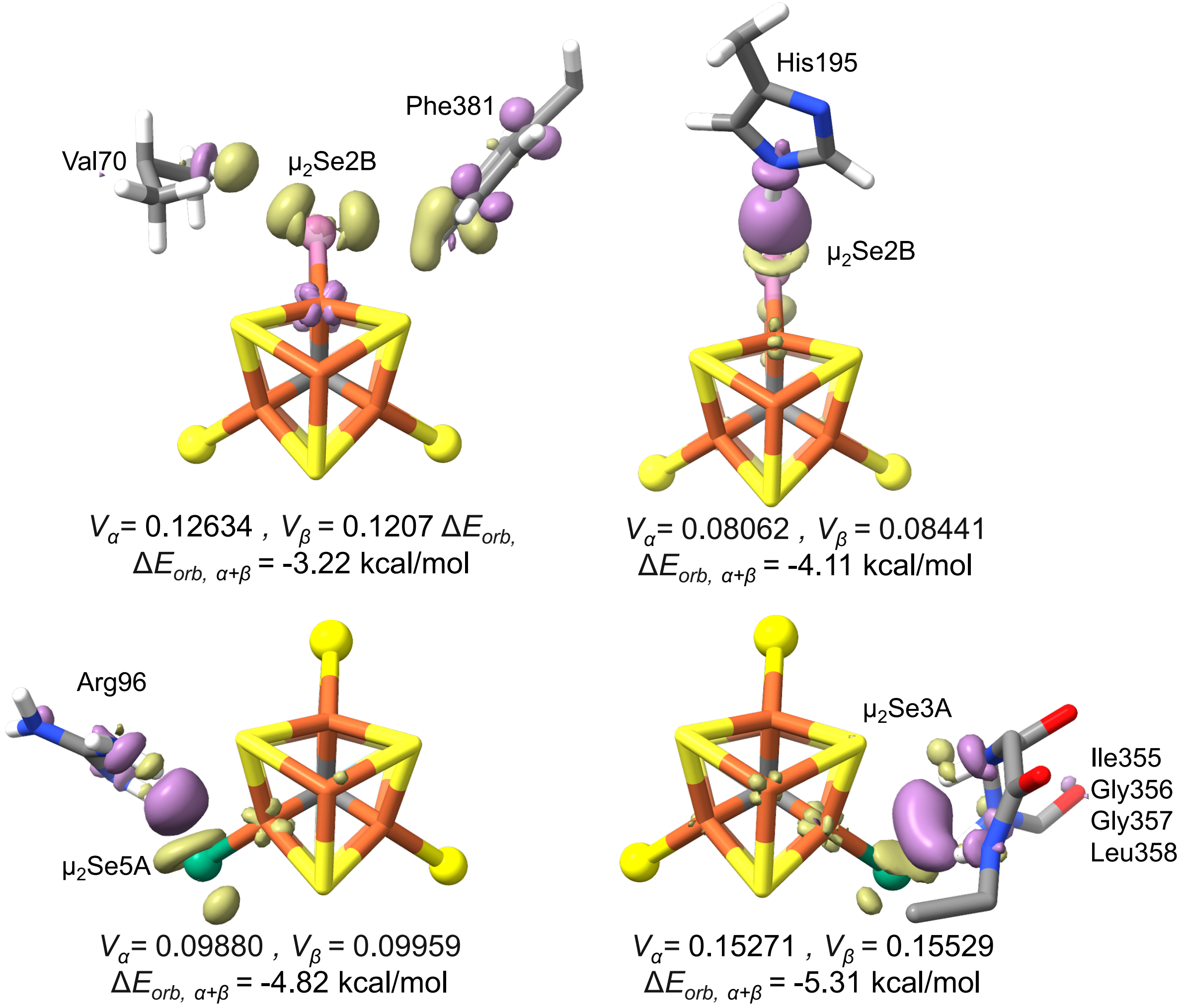

Figure S20. ETS-NOCV deformation density pairs describing the dominant orbital interactions between Se-FeMoco and the amino acid of interest. The corresponding NOCV eigenvalues (v_α, v_β) and orbital interaction energy contributions ($\Delta E_{orb,\ \alpha+\beta}$) are shown below each panel. Energy contributions are express in kcal/mol. All deformation densities are visualized at an isosurface value of 1×10^{-3} e/Bohr^3. Pale olive and lavender surfaces indicate regions of charge depletion and accumulation, respectively.

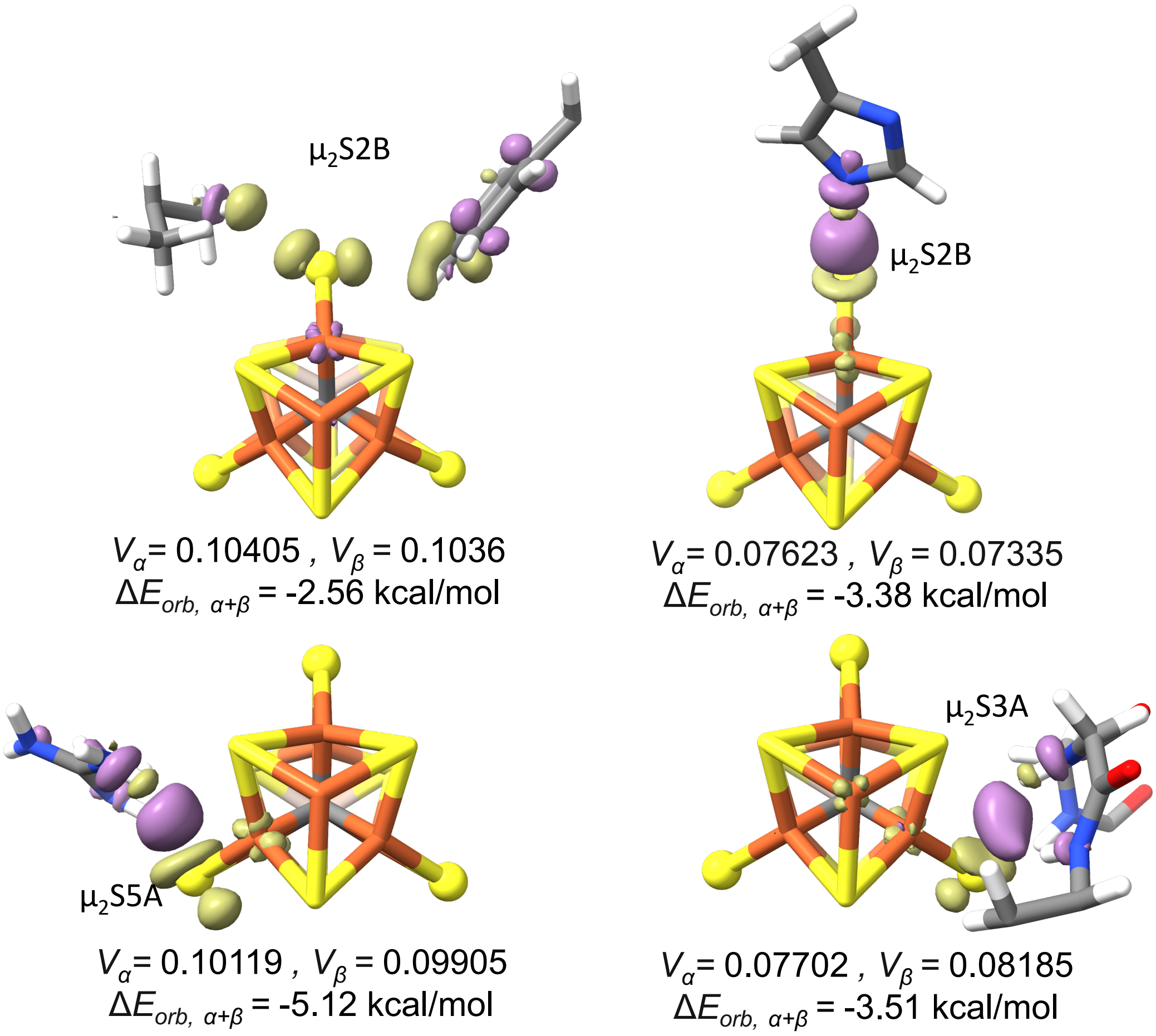

Figure S21. ETS-NOCV deformation density pairs describing the dominant orbital interactions between WT-FeMoco and the amino acid of interest. The corresponding NOCV eigenvalues (v_α, v_β) and orbital interaction energy contributions ($\Delta E_{orb,\ \alpha+\beta}$) are shown below each panel. Energy contributions are express in kcal/mol. All deformation densities are visualized at an isosurface value of 1 × 10^{-3} e/Bohr^3. green and purple surfaces indicate regions of charge depletion and accumulation, respectively.

9. Basicity Trend Among the Belt Sites

Table S14. Relative energies (kcal/mol) for μ_2-sulfide protonation at each E_0 conformer of FeMoco's resting state, calculated using the r^{2}SCAN and TPSSh functionals with protonated belt sulfides. The BS7-346 solution was applied for protonated S2B, BS7-247 for protonated S5A, and BS7-235 for protonated S3A.

	ΔE (kcal/mol)					
Functional	S2B(H)		S5A(H)		S3A(H)	
	Phe381	Val70	Arg359	Arg96	Arg359	AA
r^{2}SCAN	0	0.24	1.93	3.53	7.50	8.09
TPSSh	0	0.46	2.16	2.78	7.58	8.46

10. References

(1) Pipek, J.; Mezey, P. G. A Fast Intrinsic Localization Procedure Applicable for Ab Initio and Semiempirical Linear Combination of Atomic Orbital Wave Functions. *J. Chem. Phys.* **1989**, *90* (9), 4916–4926. https://doi.org/10.1063/1.456588.

(2) Benediktsson, B.; Bjornsson, R. QM/MM Study of the Nitrogenase MoFe Protein Resting State: Broken-Symmetry States, Protonation States, and QM Region Convergence in the FeMoco Active Site. *Inorg. Chem.* **2017**, *56* (21), 13417–13429. https://doi.org/10.1021/acs.inorgchem.7b02158.
